# Combination therapy for tuberculosis treatment: pulmonary administration of ethionamide and booster co-loaded nanoparticles

**DOI:** 10.1038/s41598-017-05453-3

**Published:** 2017-07-14

**Authors:** Joana Costa-Gouveia, Elisabetta Pancani, Samuel Jouny, Arnaud Machelart, Vincent Delorme, Giuseppina Salzano, Raffaella Iantomasi, Catherine Piveteau, Christophe J. Queval, Ok-Ryul Song, Marion Flipo, Benoit Deprez, Jean-Paul Saint-André, José Hureaux, Laleh Majlessi, Nicolas Willand, Alain Baulard, Priscille Brodin, Ruxandra Gref

**Affiliations:** 1 Univ. Lille, CNRS, INSERM, CHU Lille, Institut Pasteur de Lille, U1019 - UMR 8204 - CIIL - Center for Infection and Immunity of Lille, F-59000 Lille, France; 2 University of Paris-Sud, University Paris-Saclay, CNRS, UMR 8214 - Institute for Molecular Sciences of Orsay (ISMO), 91405 Orsay, France; 3 Univ. Lille, INSERM, Institut Pasteur de Lille, U1177 - Drugs and Molecules for living Systems, F-59000 Lille, France; 4 University Hospital Center of Angers, 49000 Angers, France; 5 Pathogénomique Mycobactérienne Intégrée, Département de Génomes et Génétique, Institut Pasteur, Paris, France

## Abstract

Tuberculosis (TB) is a leading infectious cause of death worldwide. The use of ethionamide (ETH), a main second line anti-TB drug, is hampered by its severe side effects. Recently discovered “booster” molecules strongly increase the ETH efficacy, opening new perspectives to improve the current clinical outcome of drug-resistant TB. To investigate the simultaneous delivery of ETH and its booster BDM41906 in the lungs, we co-encapsulated these compounds in biodegradable polymeric nanoparticles (NPs), overcoming the bottlenecks inherent to the strong tendency of ETH to crystallize and the limited water solubility of this Booster. The efficacy of the designed formulations was evaluated in TB infected macrophages using an automated confocal high-content screening platform, showing that the drugs maintained their activity after incorporation in NPs. Among tested formulations, “green” β-cyclodextrin (pCD) based NPs displayed the best physico-chemical characteristics and were selected for *in vivo* studies. The NPs suspension, administered directly into mouse lungs using a Microsprayer®, was proved to be well-tolerated and led to a 3-log decrease of the pulmonary mycobacterial load after 6 administrations as compared to untreated mice. This study paves the way for a future use of pCD NPs for the pulmonary delivery of the [ETH:Booster] pair in TB chemotherapy.

## Introduction

Despite the development of modern medicine, tuberculosis (TB) is still a major health problem. About one-third of the world’s population is infected with *Mycobacterium tuberculosis*, the bacterium that causes TB. According to WHO, 10.4 million people had active TB and 1.8 million people died of TB in 2015^1^. Lungs are the primary site for *M*. *tuberculosis* infection. When a patient with active TB sneezes, coughs or spits, the droplets containing bacteria can be inhaled by surrounding people who can become infected. *M*. *tuberculosis* is a facultative intracellular bacterium, able to survive and multiply inside phagocytes such as macrophages by subverting the effector functions of these important innate immune cells. *M*. *tuberculosis* can persist in the host in a dormant state for a long period of time so that the risk of reactivation, even decades after infection, exists^2^.

TB can usually be treated with a daily six months course of standard, or first-line, anti-TB drugs. If first-line drugs are misused or mismanaged, the onset of multidrug-resistant TB (MDR-TB) can occur. MDR-TB is defined as TB caused by strains that are resistant to at least isoniazid (INH) and rifampicin (RIF), two powerful first-line anti-TB drugs. MDR-TB takes up to two years of chemotherapy with second-line drugs such as ethionamide (ETH), fluoroquinolones or aminoglycosides, which are less effective and/or induce more severe side effects than the first-line drugs^3^.

Despite its usefulness, ETH exhibits reduced *in vivo* half-life and high toxicity^4^. To achieve an effective serum concentration of ETH through oral administration, doses of at least 500 mg are required^5^. However, adherence to such doses is difficult for the patients because of adverse side effects occurring mainly in the gastrointestinal tract and in the liver^4^.

ETH is a prodrug that requires bioactivation. This process is mediated by the bacterial monooxygenase EthA, which is under the control of the transcriptional repressor EthR. The relative low level of ETH bioactivation by EthA is largely responsible for the low sensitivity of *M*. *tuberculosis* to this antibiotic^6,7^. Recently, molecules inducing conformational changes in EthR, leading to the inhibition of its repressor function, have been discovered^8^. These molecules were found to considerably enhance the activity of ETH and were called “ETH boosters” or more simply “boosters”. It was observed in a TB-infected mouse model that the intraperitoneal administration of a booster in combination with an oral administration of ETH significantly increased the antimycobacterial activity of ETH, as compared with the single drug at the same dose^8^.

As lungs are the major site of *M*. *tuberculosis* infection, administration by the pulmonary route could be a valid strategy to improve the efficacy of the [ETH:booster] pair. This strategy could provide higher drug concentrations at the target site and less systemic side effects as compared to other administration routes^9–11^. Some examples are described with other anti-TB drugs such as RIF, INH and pyrazinamide (PZA), encapsulated in poly(D,L-lactic-co-glycolic acid) (PLGA)^12^ or alginate^13^ nanoparticles (NPs). In the guinea pig TB preclinical model, only few administrations of aerosolized NPs were required to reach the same effects as those obtained with free anti-TB drugs delivered daily by oral route. Nowadays, intrapulmonary aerosol delivery of anti-TB drugs is considered as a promising alternative strategy able to reduce at the same time doses, dose frequency and systemic side effects^14^.

In this context, we questioned whether the [ETH:booster] pair could be administered directly to the lungs by means of engineered NPs^15^. The use of NPs to effectively solubilise a variety of poorly soluble pharmaceutical agents, including antibiotics, is currently at the forefront of drug delivery research. However, the efficient incorporation of these molecules has to overcome technological barriers. For instance, booster molecules do not have sufficient water solubility for *in vivo* efficacy (below mg/mL)^16^ and have low affinity for most of the biodegradable materials used to prepare NPs. ETH was already encapsulated in colloidal systems^17^, microparticles^18,19^ and PLGA NPs^20^. However, the strong tendency of ETH to crystallize in aqueous environments as well as in biological fluids makes its incorporation in a nanocarrier particularly difficult^20^. To address the challenges related to the co-encapsulation of ETH with the booster BDM41906^16^ (hereby referred to as Booster), we considered investigating NPs made of biodegradable PLGA and poly(lactic acid) (PLA) copolymers and NPs of polymeric β-cyclodextrins (pCD).

PLA and PLGA (co)polymers are the most employed materials to formulate NPs because of their well-documented biocompatibility and biodegradability. Already approved by the Food and Drug Administration (FDA), they form a versatile family allowing to tune the NPs encapsulation and release properties according to their chemical composition (glycolic/lactic acid ratio) and their molecular weight (MW).

Cyclodextrins (CDs) are a family of biocompatible cyclic oligosaccharides made of α-D-glucopyranose units joined through α(1 → 4) linkages in a circular way to form a ring with a hydrophilic exterior and a hydrophobic cavity, in which many active agents can be hosted. For example, hydroxypropyl β-CD were used to solubilize the Booster, in order to assess the pharmacokinetic profile of this drug^16^. Many CD-based derivatives are widely used as delivery systems for their well-established biocompatibility in humans, due to their low toxicity and absence of immune stimulation even at high dosage^21–23^. Advantageously, polymeric CD (pCD) NPs were well-tolerated *in vivo* and were found to efficiently incorporate a series of active molecules without the need of organic solvents^24–28^.

In all cases, the NPs developed here were characterized by colloidal sizes, narrow size distributions and high incorporation efficiencies of the [ETH:Booster] pair. Interestingly, the pCD NPs allowed for an efficient one-step incorporation of both ETH and Booster by a “green” procedure, meaning without using any organic solvent.

To investigate the efficacy of drug-loaded NPs against *M*. *tuberculosis*, we used two phenotypic assays that are disease-relevant and amenable to high-throughput. The first one is a photometer-based assay relying on the monitoring of fluorescence from green fluorescence protein (GFP)-expressing *M*. *tuberculosis* strain. The second is an image-based model that allows the multi-parametric quantification of *M*. *tuberculosis* replication inside its favourite niche, the macrophage. The latter high-content imaging method has now been successfully implemented in several laboratories for drug discovery purpose^29–33^. Indeed there is more and more consensus that this technology is as reliable as CFU counting for the quantification of bacterial load and moreover, it has the advantage of giving additional information on the cytotoxicity of the studied compounds, which is here of main importance for testing NP formulations.

*In vitro* studies on *M*. *tuberculosis* H37Rv in axenic conditions as well as in infected RAW 264.7 macrophages showed that the treatment with the developed NPs containing [ETH:Booster] pair was as potent as that with free [ETH:Booster] pair solubilized in organic solvents. Among tested formulations, the pCD NPs displayed the best physicochemical characteristics and were thus selected for *in vivo* studies. For the first time, they were shown here not significantly modify the composition of lungs immune cell subsets after repeated pulmonary administration of high doses in uninfected mice. Then developed pCD NPs loaded with [ETH:Booster] were administered *via* the endotracheal route to the lungs of *M*. *tuberculosis*-infected mice using a Microsprayer® device, which enables the delivery of a precise volume of compound^34^. The formulation was able to reduce the mycobacterial load using subtherapeutic doses in a short course of infection model. With a two weeks treatment, the mycobacterial load was drastically decreased by 3-logs as compared to the non-treated animals.

## Results and Discussion

To address the challenge of treating *in vivo M*. *tuberculosis* with the [ETH:booster] pair, biodegradable PLA and PLGA NPs and “green” pCD NPs were studied here for their ability to circumvent the bottlenecks related to the limited water solubility of the booster (below mg/mL) and the strong ETH tendency to crystalize. The booster used in this study was the recently developed booster BDM41906^16^ (hereby referred to as Booster).

### Biodegradable PLA/PLGA NPs

In the first part of the study, a series of PLA/PLGA NPs containing [ETH:Booster] were prepared by nanoprecipitation, a simple one-step encapsulation method. Briefly, a dimethyl sulfoxide (DMSO) solution of PLA or PLGA and drug(s) was precipitated in injectable water leading instantaneously to the production of NPs without the need of any surfactant. DMSO was chosen because, among the pharmaceutically acceptable organic solvents, it is the only common solvent for ETH, Booster and polymers. Immediately after pouring the organic solution in water, monodisperse NPs with a mean diameter of around 170 ± 10 nm were obtained. Remarkably, the NPs mean diameter was stable upon storage at room temperature for up to 5 months with only less than 5% size variation (Supplementary Fig. S1).

The major challenge was to avoid ETH crystallization during NPs storage. Whatever the (co)polymers and the experimental conditions used, the highest ETH loadings obtained without crystallization were only about 11 weight percent (wt%) (for details see Supplementary Figs S2 and S3, Section I). Unfortunately, co-incorporation of Booster in the NPs further decreased the ETH drug loading (DL) (see Supplementary Information, Section I).

A similar strong tendency of a drug to form crystals has been previously observed by Layre *et al*.^35^, when encapsulating busulfan in biodegradable poly(alkyl cyanoacrylate) NPs. The busulfan free crystals in the NPs suspensions were extremely difficult to be removed. The study highlighted the challenges related to the encapsulation and consequent delivery of crystalline drugs *in vivo*^36^. Therefore, to simultaneously avoid ETH crystallization and to increase the [ETH:Booster] DL, an alternative method for the preparation of PLA NPs was investigated. PLA NPs made of P4 were prepared by nanoemulsion method using polyvinyl alcohol (PVA) as surfactant.

A dramatic increase of the ETH loading efficiency was observed as compared to the nanoprecipitation method (Table 1). In particular an ETH DL of 38 ± 2 wt% corresponding to an encapsulation efficiency (EE) of 77 ± 5 wt% was found.

**Table 1:** Main characteristics of empty and drug-loaded PLA NPs made of P4 by nanoemulsion.

Formulation	Size distribution (PCS)		ETH Content		Booster Content	
	Mean diameter (nm ± SE)	PdI	DL (wt% ± SD)	EE (wt% ± SD)	DL (wt% ± SD)	EE (wt% ± SD)
PLA NPs	254 ± 4	0.054	—	—	—	—
PLA NPs [ETH]	267 ± 3	0.074	38 ± 2	77 ± 5	—	—
PLA NPs [ETH:Booster]	274 ± 4	0.090	36 ± 4	76 ± 5	26 ± 3	51 ± 8
PLA NPs [Booster]	277 ± 5	0.075	—	—	23 ± 2	46 ± 2

NPs mean hydrodynamic diameters were determined by PCS and are reported as Mean diameter (Z average nm ± standard error) and Polydispersity Index (PdI). ETH and Booster drug loading (DL) and encapsulation efficiency (EE) were determined by LC-MS-MS and RP-HPLC (the data are mean values ± standard deviation).

Remarkably, ETH and Booster could be well co-incorporated in the same NPs. PLA NPs were characterized by an EE of ETH and Booster of 76 ± 5 wt% and 51 ± 8 wt%, respectively (Table 1). Interestingly, the presence of both drugs in the formulation did not significantly influence the mean diameter of the NPs (Table 1). The NPs size distribution was evaluated by using three independent techniques: photon correlation spectroscopy (PCS), NP tracking analysis (NTA) and transmission electron microscopy (TEM). The NPs mean diameter using PCS, employing dynamic light scattering (DLS) technique, ranged from 254 to 277 nm (Table 1). Polydispersity index (PdI) was less than 0.1, indicating the formation of monodisperse NPs. The Zeta Potential (ZP) of the different formulations was −5 ± 0.5 mV, for both drug loaded or unloaded NPs.

The second method, NTA, has the main advantage of tracking NPs individually in their Brownian motion but it needs a high dilution of NPs suspensions. It can determine at the same time the hydrodynamic diameter of NPs and their concentration in various media^37^ (Supplementary video S1). The mean diameter of PLA NPs determined by NTA ranged from 171 to 180 nm and the NPs concentrations were of around 1 × 10^9^ particles/mL (after dilution by a factor of 10,000) with no significant differences between NPs loaded or not with drug(s). These values are smaller than the ones obtained by PCS (Supplementary Table S1). The mean diameters obtained by PCS are often overestimated, due to the presence of aggregates or to the fact that the largest NPs scatter more light than the smaller ones^38,39^. In this study, NTA was the most adapted technique to estimate the average hydrodynamic diameters by individually tracking the NPs in suspension, while DLS was a sensitive and adapted method for stability studies showing that no aggregates could be detected in PLA NPs prepared by nanoemulsion.

**Figure 1:**
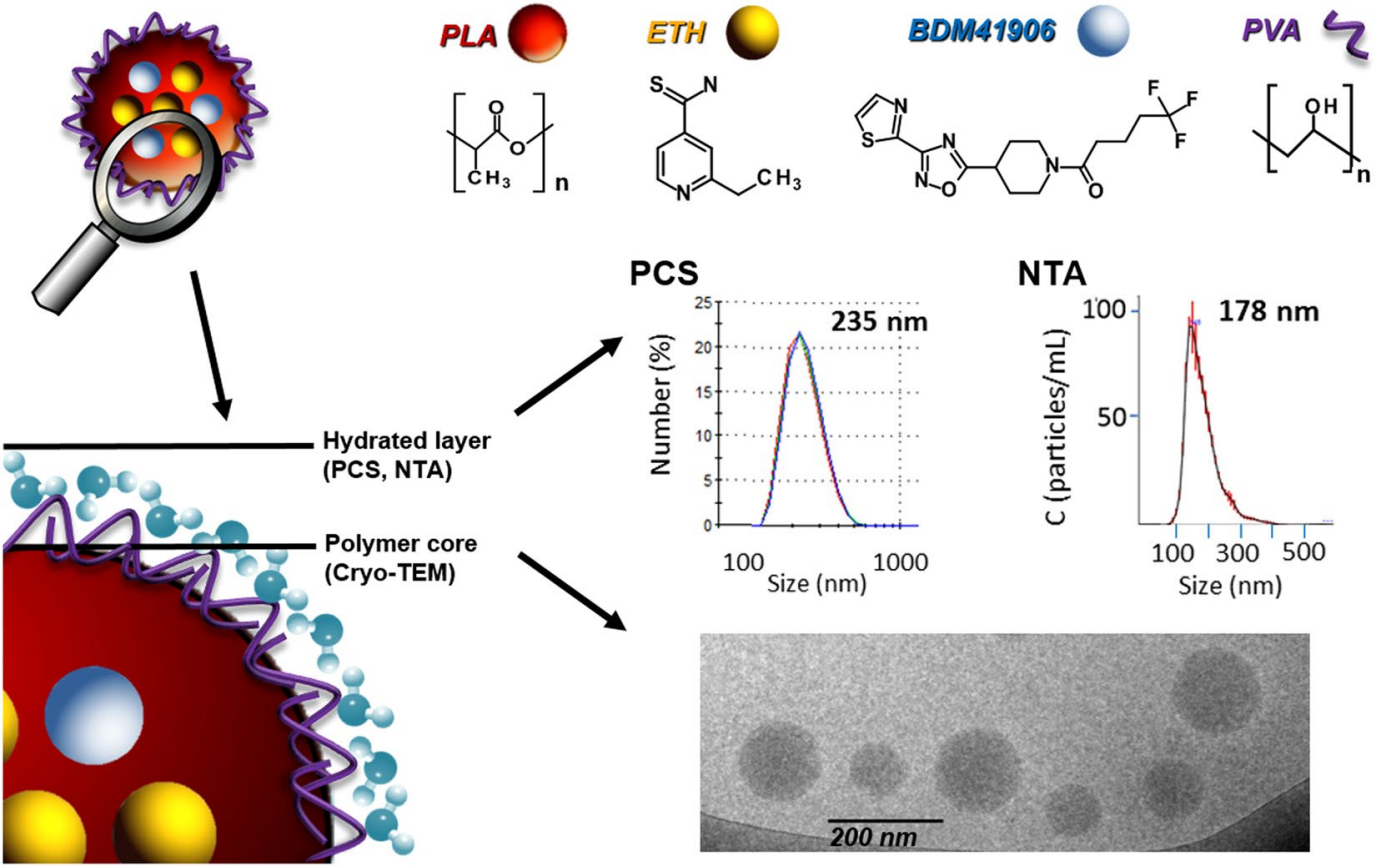
Chemical structure of PLA NPs components (nanoemulsion technique) and characterization of NPs mean diameter using three independent methods, PCS (number average data), NTA and Cryo-TEM. Typical example of NPs encapsulating [ETH:Booster]. Schematical representation of PLA NPs structure and components. Cryo-TEM allows determining the dry diameter (polymer core), whereas PCS and NTA allow measuring the hydrodynamic diameter (Polymer core + hydrated layer).

Furthermore, in order to get more in-depth information about the size and the morphology of the NPs, cryogenic TEM (cryo-TEM) analysis was performed (Fig. 1). Typical images of [ETH:Booster]-loaded PLA NPs (Fig. 1) showed spherical shapes and homogenous structures. The morphology of the NPs was not influenced by the presence of the drugs.

Besides, a statistical evaluation of the size distribution was performed on more than 2,000 NPs, showing a mean diameter of about 100 nm ± 50 nm (Supplementary Table S1). The mean diameter obtained by cryo-TEM was significantly lower from the ones obtained by both NTA and PCS (Supplementary Table S1). Indeed, the diameters determined by cryo-TEM correspond to the dry diameter of the NPs, without taking into account the hydrated PVA shell which is flattened during the observations^40,41^. Indeed, it has been reported that PLA and PLGA NPs possess a hydrated PVA shell of several nm thickness. This could explain the difference of around 70 nm in sizes found between the mean diameter obtained by NTA and the one obtained by cryo-TEM (Supplementary Table S1).

The different methods used to evaluate the NPs size distribution are summarized in Fig. 1, highlighting the importance of using multiple and complementary techniques to fully characterize the NPs systems^42^. The preparation of PLA NPs by nanoemulsion proved to be particularly advantageous for the co-encapsulation of the [ETH:Booster] pair as compared to nanoprecipitation. Both methods led to the formation of NP suspensions stable upon storage in terms of size and PdI (Supplementary Fig. S4). However ETH crystallisation could not be totally avoided even by the nanoemulsion method as the formation of tiny crystals was observed during storage.

### “Green” pCD-based NPs containing [ETH:Booster] pair

**Figure 2:**
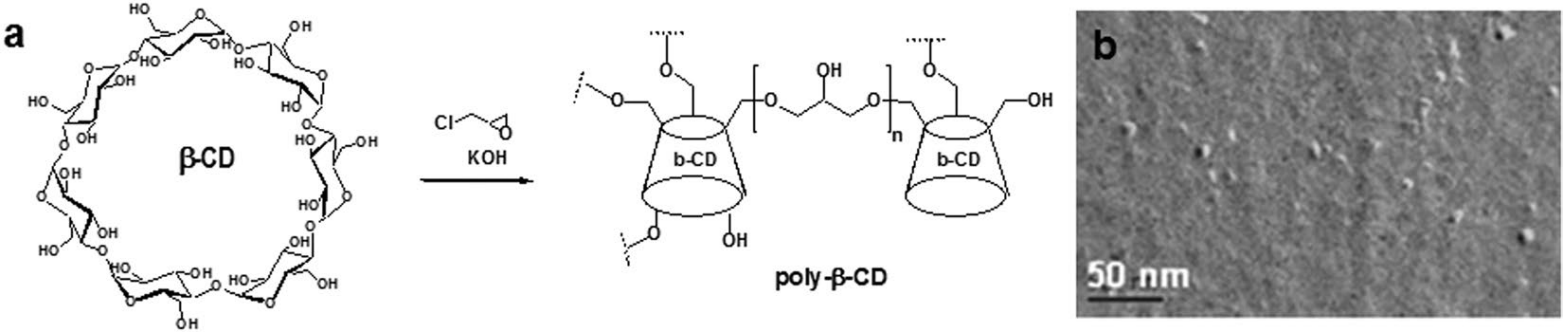
Nanoparticles made of pCD. (**a**) Polymerisation of β-CD with epichlorohydrin leading to the formation of cross-linked poly(2-hydroxy-1,3-propylenedioxy)-polycyclodextrin polymer (pCD) and (**b**) TEM image after freeze fracture of pCD NPs (mean size around 10 nm).

To overcome the technological challenge of completely avoiding ETH crystallisation, a different approach was attempted by encapsulating this drug at a molecular level. To this end the use of polymeric CDs was particularly appealing. NPs made of cross-linked poly(2-hydroxy-1,3-propylenedioxy)-polycyclodextrin polymer (pCD) were prepared according to previous reports^28^ by polymerization of β-CD with epichlorohydrin in alkaline medium (Fig. 2a). We report here for the first time their use to co-encapsulate drugs, together with their pulmonary application.

TEM micrographs after freeze-fracture revealed spherical shapes and diameters of around 10 nm (Fig. 2b). However, DLS and NTA methods were inappropriate to characterize the pCD NPs because of their small sizes. Remarkably, the pCD NPs could be concentrated up to 200 mg/mL without aggregation and were able to soak out both ETH and Booster from their aqueous solutions in the absence of organic solvents. This is a major advantage as compared to PLA and PLGA NPs, which could not be concentrated without aggregation to more than 5 mg/mL and for which ETH and Booster incorporation requires the use of organic solvents.

The aqueous solubility of both drugs was enhanced as a function of the pCD concentration. In the absence of pCD, the aqueous solubility of ETH determined by high performance liquid chromatography (HPLC) was found to be only 0.48 mg/mL. After 2 days of incubation at room temperature with pCD NPs at a concentration of 50 mg/mL, the apparent solubility of ETH was considerably increased to 1.5 ± 0.2 mg/mL, because of the affinity of the lipophilic drug molecules for the hydrophobic microenvironment of the pCD NPs. Furthermore, ETH solubility was found to be proportional with the pCD NPs concentration. For example, pCD NPs at a concentration of 200 mg/mL increased by 10 fold the apparent solubility of ETH to 4.9 ± 0.6 mg/mL.

Moreover, pCD NPs were highly efficient to incorporate the slightly water-soluble Booster. In particular, pCD NPs at a concentration of 200 mg/ml were able to incorporate up to 5 mg/mL of both ETH and Booster. It is worth mentioning that in order to solubilise the same amount of ETH, a mixture of DMSO: water containing as high as 40–50 vol% DMSO is needed. This elevated amount of organic solvent is incompatible with an *in vivo* administration.

In all cases, pCD NPs containing ETH alone or in combination with Booster were stable upon storage without the presence of any aggregates and/or the formation of ETH crystals (for further details see Supplementary Information - Section I). These results highlight the interest of this “green” strategy based on confining both drugs in a molecular form inside the NPs, totally avoiding the risks related to the use of organic solvents. Indeed, insoluble drugs were shown to readily accommodate in the hydrophobic CD cavities as well as in the confined micro-domains in the crosslinked pCD NPs without crystallization^25^.

### Efficacy of the NPs containing [ETH:Booster] pair against *M*. *tuberculosis* replication in phenotypic extracellular and intracellular assays

**Figure 3:**
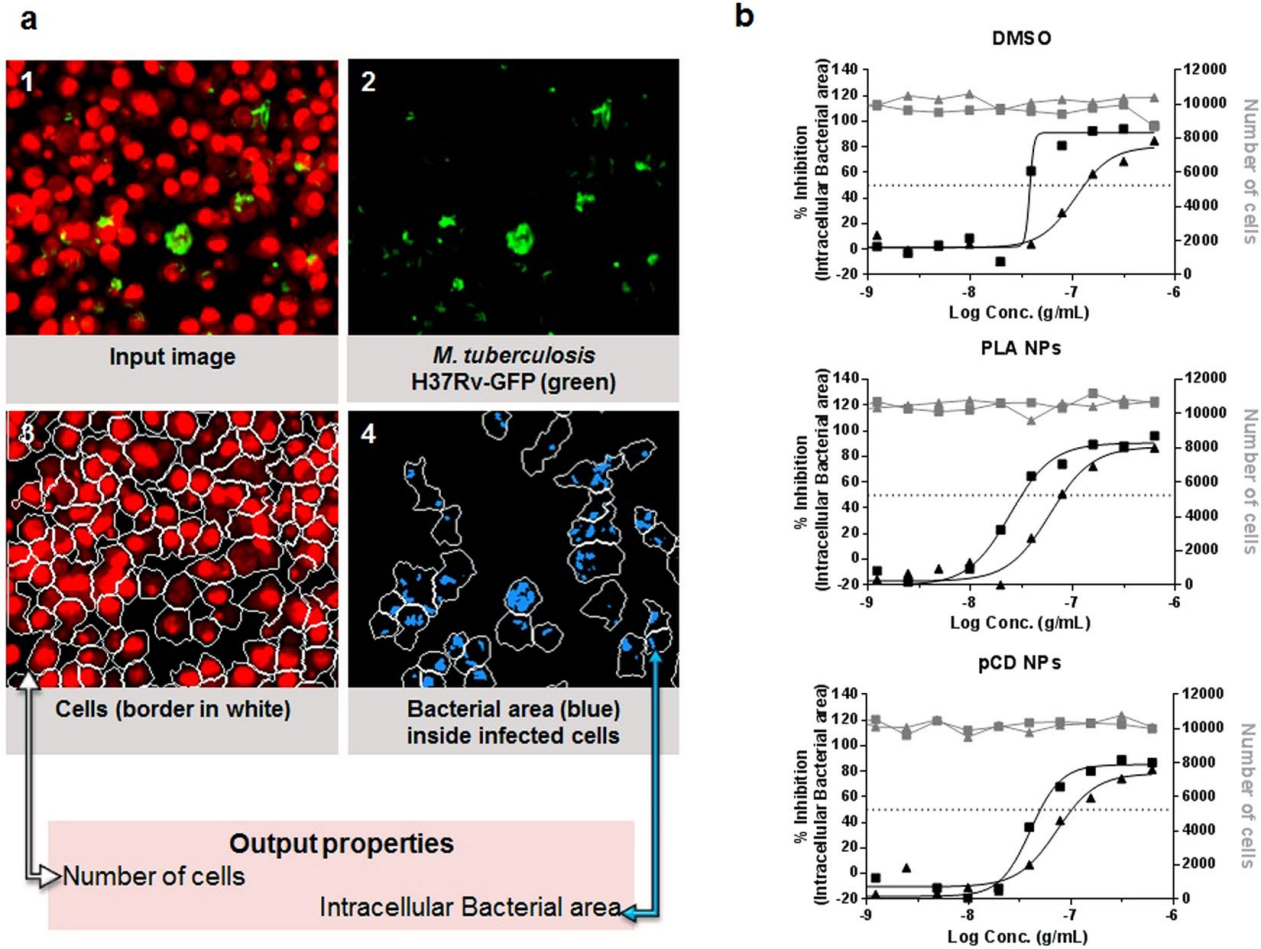
Intracellular antitubercular activity. (**a**) Scheme representing image-based analysis method performed with the image-analysis software Columbus 2.5.1 (PerkinElmer). **1**. Input image: cell nuclei and cell cytoplasm were stained with the DNA dye Syto60 and detected in the red channel; *M*. *tuberculosis* H37Rv-GFP was detected in the green channel. **2**. *M*. *tuberculosis* H37Rv-GFP detected in the green channel. **3**. Cells detection in the red channel. The estimated cell limit is represented in white. **4**. Bacterial area detected inside the cells area is represented in blue. Data related to the area of cells was detected in the red channel and data related to the bacterial area was detected in the green channel. (**b**) % of inhibition of intracellular bacterial area (black points) and number of cells (grey points) obtained with ETH (solid triangle) and [ETH:Booster] (solid square) in DMSO, PLA NPs and pCD NPs. Data presented corresponds to one representative experiment of three experiments.

PLA NPs prepared by nanoemulsion and pCDs NPs that contain [ETH:Booster] pair were then investigated for their activity against *M*. *tuberculosis* replication. To monitor the activity, we used two phenotypic assays^29,43^ that rely on the use of a fluorescent strain of *M*. *tuberculosis* H37Rv (H37Rv-GFP) and RAW 267.4 macrophages. Briefly, a series of two-fold dilutions of [ETH:Booster] NPs were first performed and 5 µL of each dilution added into a 384-well microplate. It is worth noting that with this method, both ETH and Booster are diluted simultaneously. For DMSO vehicle controls (ETH and Booster) different volumes of solution were transferred using a nanoliter acoustic dispenser and backfilled with DMSO to 500nL, allowing the use of a minimum volume of DMSO that is toxic above 1% final concentration. To obtain the same volume for [ETH:Booster] NPs and [ETH:Booster] DMSO diluted samples, cell medium was added up to 5 µL in the latter ones. Then, 45 µL of PBS-washed H37Rv-GFP bacteria were added alone for the extracellular test and 45 µL of the same bacteria that were previously incubated with RAW 267.4 macrophages for 2 hours were added for the intracellular test. After 5 days incubation at 37 °C, 5% CO_2_, the amount of bacteria was quantified by acquisition of the GFP-fluorescence signal on a multimode reader for the extracellular assay and by confocal fluorescence microscopy and automated image-analysis for the intracellular assay (Fig. 3a, Supplementary Fig. S5).

**Figure 4:**
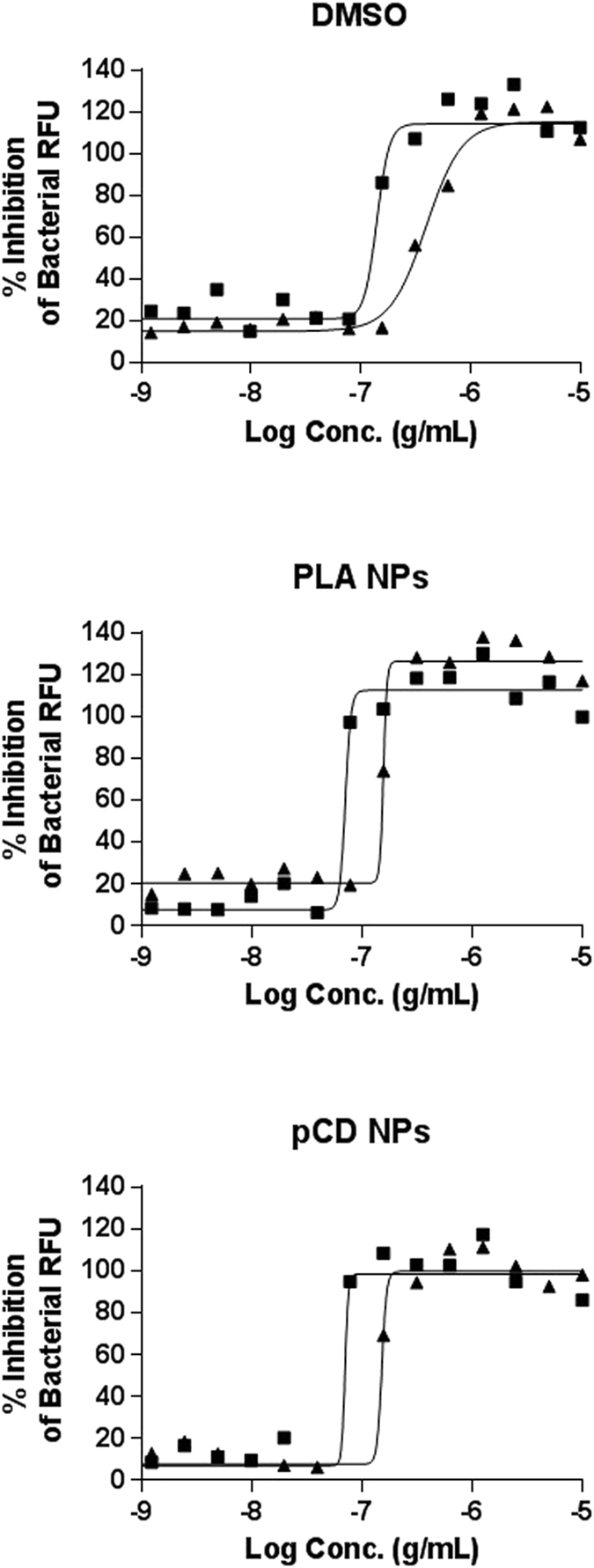
Extracellular antitubercular activity. % of inhibition on bacterial RFU obtained with ETH (solid triangle) and [ETH:Booster] (solid square) in DMSO, PLA NPs and pCD NPs. Data presented correspond to one representative experiment of three experiments.

For both assays and each of the combinations, dose-response curves for the different parameters were generated after normalization on DMSO-negative and INH-positive control (Fig. 3b and Fig. 4). For the image-based assay, the intracellular bacteria area was used as a correlate of intracellular growth, while the total cell number is an indicator of the cytotoxicity of the compounds (Fig. 3b). All samples, free-DMSO and encapsulated ETH and [ETH:Booster] pair showed a dose-dependent inhibition and there was no cytotoxicity for any of the samples. For both assays, the results were then further summarized as the concentration of ETH required to inhibit 50% of the bacterial growth (IC_50_) (Table 2).

**Table 2:** Efficiency of the different formulations against *M*. *tuberculosis* H37Rv-GFP extracellular or intramacrophage replication.

Formulation		Extracellular assay *IC50* (*µg/mL*) ± *SD*	Intracellular assay *IC50* (*µg/mL*) ± *SD*
Controls (DMSO)	ETH	0.30 ± 0.10	0.11 ± 0.01
	[ETH:Booster]	0.11 ± 0.06	0.06 ± 0.02
PLA NPs (Nanoemulsion)	PLA::ETH	0.12 ± 0.04	0.09 ± 0.05
	PLA::[ETH:Booster]	0.06 ± 0.02	0.04 ± 0.02
pCD NPs	pCD::ETH	0.13 ± 0.04	0.06 ± 0.01
	pCD::[ETH:Booster]	0.06 ± 0.02	0.04 ± 0.01

Results, expressed as concentration of ETH required to inhibit 50% of the bacterial growth in µg/mL (IC_50_), were calculated by nonlinear regression analysis using the equation for a sigmoidal dose-response curve with variable slope using the GraphPad Prism 5.0 software. Results are shown as the mean ± standard deviation of three independent experiments.

As expected, the IC_50_ for [ETH:Booster] pair was lower than that of ETH alone in DMSO conditions and within the range of our previous report given the variability of the assay^16^. Next, the ETH that has been formulated in PLA NPs and pCDs NPs was as potent as the ETH solubilised in DMSO, clearly demonstrating that the encapsulation of ETH was not affecting its efficiency against *M*. *tuberculosis* replication. Finally, the [ETH:Booster] pair had a two-fold increased effect in both assays compared to the parent ETH thus indicating that a synergic [ETH:Booster] combination can be efficiently reached. Also we verified that empty PLA NPs and pCD NPs had no effect on intracellular bacteria *per se* (Supplementary Fig. S6). Therefore the inhibitory effect of loaded NPs was only due to the effective delivery of their payload of ETH and Booster. These results also demonstrate the lack of toxicity of both empty and loaded NPs (Supplementary Fig. S6b, Fig. 3b).

### Effect of NPs containing [ETH:Booster] pair via endotracheal aerosol administration against a short-course *M*. *tuberculosis* challenge

Given the satisfactory results mentioned above, we decided to investigate the antitubercular properties of the NPs in an *in vivo* model of TB infection. To this end, the NPs co-loaded with ETH and Booster were administered to infected mice via pulmonary administration.

Different devices, such as whole body exposure chamber, head and nose-only exposure systems, deposition by tracheotomy and Microsprayer® have been evaluated to administer NPs directly in the lungs of *M*. *tuberculosis* infected mice^34,44,45^. The currently available systems for pulmonary administration in small animals have several limitations such as imprecisions due to the difficulty of measuring the amount of inhaled drugs, delivery of low fraction of the administered drug or invasive surgery^44,45^. Stemming from these considerations, the choice of the appropriate delivery device is of outmost importance. The Microsprayer® device appeared as the most reliable device for precise pulmonary administration to rodents as it has been shown that up to 89 ± 10% of the administered dose was efficiently delivered to mouse lungs^46–48^. Moreover, the use of Microsprayer® was shown to allow an efficient bilateral distribution of the NPs in the lungs^49^. However, not all drug formulations are compatible with this delivery system. Loaded PLA NPs presented a high tendency to be retained by the Microsprayer® and the ETH content from each spray was highly variable, compromising the study of their *in vivo* efficacy.

On the contrary, the pCD NPs could be efficiently and reproducibly administered *in vivo* using the Microsprayer®. Indeed, the average amount of pCD NPs suspension (50.9 ± 0.2 mg) delivered by the Microsprayer® was very close to the expected one (52.0 ± 0.2 mg). The weight of each spray was constant, fully reproducible and the standard deviation was low. Remarkably, 98 ± 0.7 wt **%** of the NPs suspension was recovered after the passage though the Microsprayer®. In addition, the drug loading of pCD NPs was determined by HPLC before and after the passage through the device. For instance, for a suspension of 100 mg/mL of pCD NPs loaded with 2 mg of ETH a recovery of 99 ± 4% was obtained.

**Figure 5:**
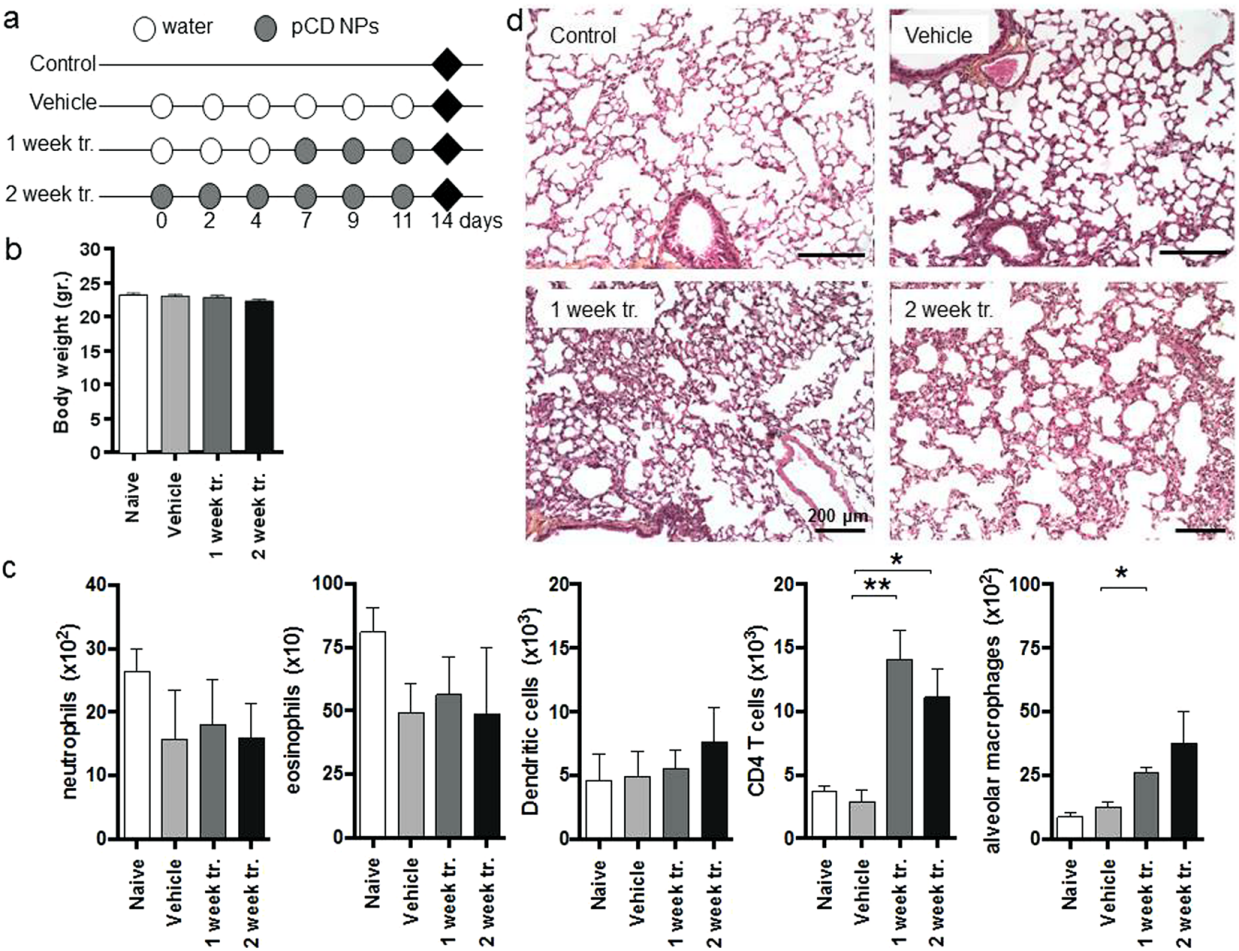
Impact of pCDs NPs administration on pulmonary homeostasis in mouse. (**a**) Protocol. Water (Vehicle) or nanoparticles were administered to mice via the endotracheal route during one (1 week tr.) or two weeks (2 week tr.); ♦ = mice euthanasia. (**b**) Body weight of mice after each treatment. (**c**) Analysis of the recruitment of immune cells in the lungs parenchyma by flow cytometry. The data represent the number of selected cells on 500,000 events; To define the different cells populations we used the following phenotypes: neutrophils (CD11b^+^GR1^+^), eosinophils (F4/80^+^SiglecF^+^), CD4 T cells (CD3^+^CD4^+^), alveolar macrophages (CD11c^+^F4/80^+^) and dendritic cells (CD11b^+^CD11c^+^); the Data are mean values ± standard deviation of one representative experiment of two independent experiments; *p < 0.1, **p < 0.01. (**d**) After treatment, lungs were analysed by histology (H-E staining, scale bar: 200 μm).

Although as already mentioned CDs are considered biocompatible, few data are available on their pulmonary toxicity. Prior early studies assessed the bronchial responsiveness, airway inflammation and histological changes upon the administration of CDs in animals *via* the pulmonary route^50,51^. These studies revealed only minor toxicity in lungs, which prompted us to study the composition of the immune cell subsets in mouse lungs following Microsprayer® administration of pCD NPs. Thus, uninfected mice were repeatedly given with aerosolized water (“vehicle”) or NPs during a period of time up to two weeks (Fig. 5a). This is the first time that high doses of pCD NPs were repeatedly administered into mouse lungs. After treatment, the potential toxicity of pCD NPs endotracheal administration was studied by analyzing the mouse body weight, lung histology and cell recruitment (Fig. 5b,c,d). Body weight was maintained during the treatment and no difference was observed for eosinophils, neutrophils and dendritic cells between the group “vehicle” and the groups that received the treatment during 1 or 2 weeks. Both the histology and the flow cytometry analyses pointed out an increase in CD4 T-cell infiltrates, which could be correlated to what has been previously reported for other CDs derivatives^51^. Also, a mild increase in the number of the alveolar macrophages was noticed upon treatment. Altogether, these results suggested that repeated treatment does not result in major modification of the composition of the immune cell subsets.

Subsequently, the ability of the pCD NPs co-encapsulating the [ETH:Booster] pair to reduce the pulmonary mycobacterial load was investigated in a mouse model of acute *M*. *tuberculosis* infection. Seven days after infection, mice received 3 doses of pCD containing [ETH:Booster] pair at 10 mg/kg for each drug by endotracheal aerosol administration using the Microsprayer® every 2 days. It is important to point out that free drugs could not be administered by the same way as controls because their solubilisation requires the use of organic solvents, incompatible with pulmonary administration. Indeed, as previously shown, up to 40–50 vol% DMSO in water is needed to solubilise equivalent amounts of drugs.

**Figure 6:**
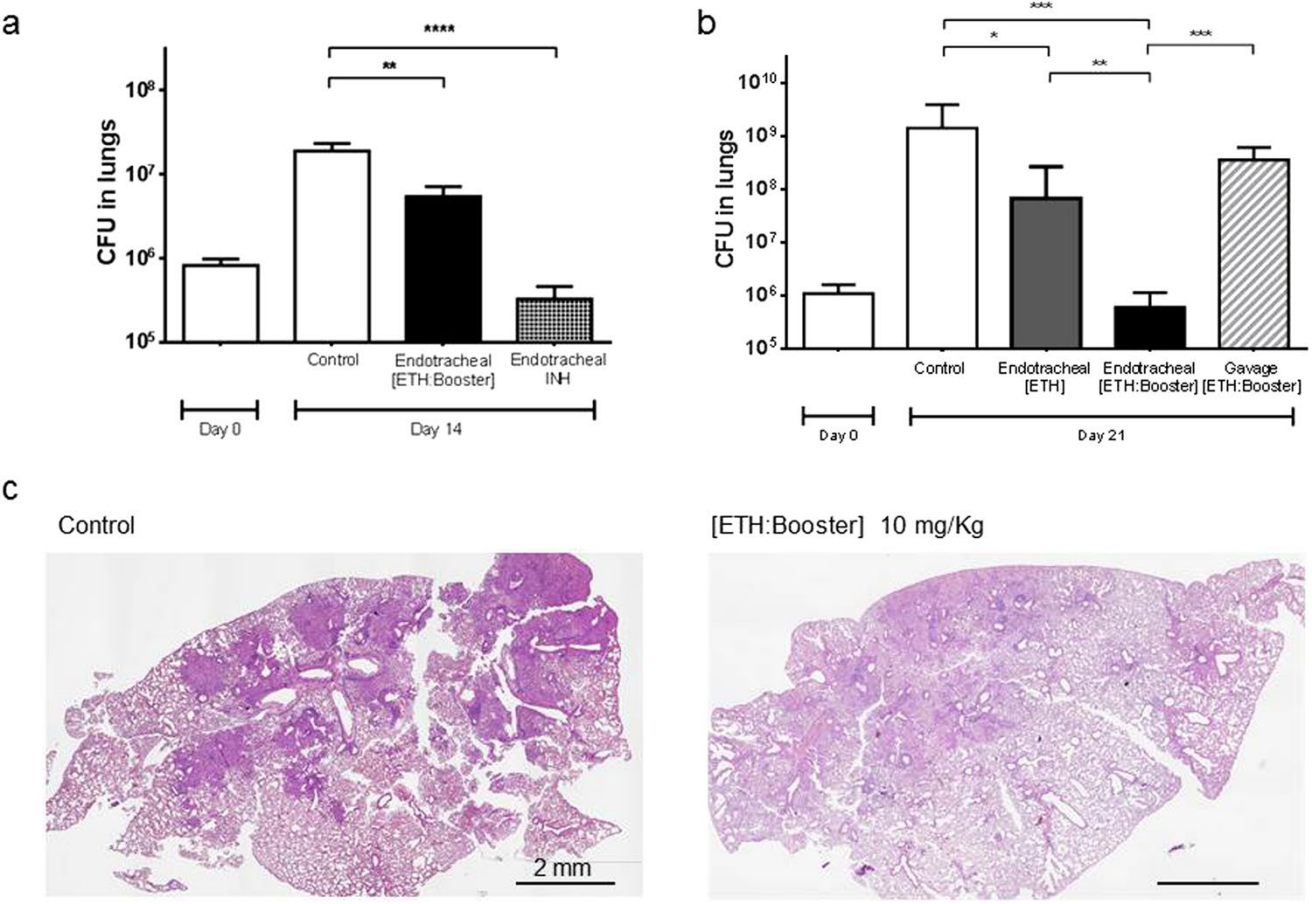
Effect of [ETH:Booster] loaded pCD NPs in a mouse model of *M*. *tuberculosis* infection. Intranasal infection was performed with *M*. *tuberculosis* H37Rv. (**a**) After 7 days mice were aerosolized endotracheally either with isoniazid (INH, 25 mg/Kg) as positive control or drug loaded nanoparticles (10 mg/Kg) three times during one week. At day 14 post-infection, mice were euthanized and CFU in the lungs were determined. Control: non-treated *M*. *tuberculosis* infected mice. Data are mean values ± standard deviation of one representative experiment of three independent experiments; **p < 0.01, ***p < 0.001. (**b**–**d**) After 7 days mice were aerosolized with drug loaded nanoparticles or received drug loaded nanoparticles by gavage six times during two weeks. At day 21 post-infection, mice were euthanized and lung infection was analysed by CFU determination and by histology (H-E staining, scale bar: 2 mm). Control: non-treated *M*. *tuberculosis* infected mice. Data are mean values ± standard deviation; *p < 0.1, **p < 0.01, ***p < 0.001.

For this reason, the water-soluble first-line INH was used as control. Compared to untreated mice, the administration of the drug loaded pCD NPs lead to a significant decrease (p < 0.01) of the pulmonary bacterial load, determined as the average number of colony forming units (CFU) (Fig. 6a). These data show that it was possible to obtain an antibacterial effect using just 3 doses of the [ETH:Booster] pair within a one week administration.

To investigate whether a longer course of treatment could lead to a greater efficacy, mice were treated with a regimen of 6 doses given over a two-week time course. As expected for this acute phase of the *M*. *tuberculosis* infection, the bacterial load for the control group increased, reaching 10^9^ CFUs (in comparison to one week treatment, 10^7^ CFUs) (Fig. 6a,b). Lungs from the mice that were treated with 6 doses of the [ETH:Booster] pCD NPs had a mean value of 5 × 10^5^ CFUs, which indicates a dramatic (3-logs) improvement in the efficacy of the formulation compared to three doses only. Corroborating with the massive decrease in the mycobacterial load, the lungs that received [ETH:Booster] pCD NPs displayed much less cell infiltrates than control animals (Fig. 6c). Moreover, the reduction of bacterial load in the lungs was much higher when ETH was co-encapsulated with the Booster (p < 0.001) than when ETH was administered alone (p < 0.1) (Fig. 6b). Noteworthy, when the formulation containing [ETH:Booster] was given by oral gavage using the same regimen (i.e. 6 doses every 2 days), the mycobacterial load in the lungs was similar as the one from non-treated controls, clearly demonstrating the benefit of using the pulmonary route for ETH and Booster co-loaded nanoparticles delivery (Fig. 6b).

Moreover, it is worth mentioning that the effect obtained in the present work with the administration of ETH:Booster 3 times/week during 2 weeks via the endotracheal route is equivalent to the one observed previously upon a daily, 6 days/week administration of ETH by gavage during 3 weeks^8^. Because it drastically reduces the dose and the frequency of the treatment, the present approach has the potential to diminish the systemic side effects of ETH.

The pulmonary delivery of pCD NPs effectively encapsulating the [ETH:Booster] pair could be a promising strategy to improve the standard therapeutic protocol in humans leading to a decrease of the typical daily dose (from 500 mg orally) of ETH^5^.

## Conclusions

The [ETH:Booster] pair was co-encapsulated in biodegradable polymeric NPs overcoming the bottlenecks inherent to the strong tendency of ETH to crystallize and the limited water solubility of Booster. Advantageously, pCD NPs effectively prevented ETH crystallization both during encapsulation and NPs storage. For the first time, an automated confocal high-content screening platform was used to evaluate the efficacy of the NPs on *M*. *tuberculosis* infected cells. Noticeably, the drugs maintained their activity after incorporation in NPs.

Among the formulations developed, pCD NPs displayed the best physicochemical properties for the simultaneous delivery of [ETH:Booster] pair in the lungs.

Moreover, pCD NPs were demonstrated not to induce main modifications of the composition of the immune cell subsets following repeated administrations at high doses *in vivo*. The treatment with 6 doses of pCD NPs containing the drug combination, led to a 3 log decrease in lungs CFU in comparison to untreated animals. Noticeably, when administering the same doses by gavage no antibacterial effect could be observed.

The pulmonary administration to animals is very challenging, as the experimental protocols have to take into account the limitations of the available tools and methods. On the other hand, the pulmonary administration in humans (metered dose inhaler) does not deal with the technical limitations related to the delivery of NP formulations in rodents, as the commercial devices allow self-administration without stress or pain.

These findings suggest further *in vivo* investigations of the developed pCD formulation. In the future, our “green” incorporation strategy could be used as a supplement to the standard treatment to contain the *M*. *tuberculosis* pulmonary manifestations and to prevent its dissemination.

Moreover, given the fact that the current regimen for TB consists in a cocktail of four drugs, the approach used here could be extended to the encapsulation of more than two drugs, simplifying the treatment, decreasing the systemic side effects and increasing the patients’ compliance to limit drug misuse.

## Materials and Methods

### Chemicals

PLGA 75:25 acid terminated (P1) (MW: 37–84 KDa, 10P002), PLGA 50:50 ester terminated (P2) (MW: 70–100 KDa, 10P016) and PLGA 50:50 acid terminated (P3) (MW: 5–20 KDa, 10P019) were kindly provided by PCAS (Expansorb, Aramon, France). PLA ester terminated (P4) (MW:10–18 kDa), PVA (87–90% hydrolysed), DMSO-99.5%, ETH and amikacin were all purchased from Sigma-Aldrich. Injectable water was purchased from Cooper (Melun, France). The solvents were of analytic grade.

The Booster named BDM41906 (5,5,5-Trifluoro-1-[4-(3-thiazol-2-yl-1,2,4-oxadiazol-5-yl)piperidin-1-yl] pentan-1-one) was synthesized as previously described^16^.

β-CD was kindly supplied by Roquette, Lestrem, France. pCD NPs of around 10 nm were produced as previously described^28,52^ by crosslinking β-CD under strongly alkaline conditions with epichlorohydrin (EP). Briefly, 100 g of anhydrous β-CD were solubilized overnight in 160 mL of NaOH 33% w/w solution. After adding 81.52 g of EP the reaction was stopped using acetone in the vicinity of the gelation point. The β-CD NPs, recovered by ultrafiltration followed by freeze-drying, contained 70% w/w β-CD, as determined by ^1^H NMR spectroscopy.

### Nanoparticle preparation

#### Nanoprecipitation

Encapsulation of ETH in PLA and PLGA NPs was carried out adapting a previously reported procedure^53^. Briefly, 0.4 mL of DMSO solution containing 10 mg polymer (PLA or PLGA) and ETH (0.5 to 7 mg) were poured drop by drop in 7 mL of injectable water under magnetic stirring. In the case of co-encapsulation, 3 mg of Booster were added to the DMSO solution.

#### Nano-emulsion

Preparation of PLA NPs was carried out as previously reported by Kumar *et al*. with slight modifications^20^. Briefly, 10 mg of ETH were solubilized in 0.2 mL of MeOH and mixed to 1.5 mL of a dichloromethane (DCM) solution containing 20 mg of PLA and 10 mg of Booster. This organic phase was poured into 4 mL of injectable water containing 0.5% w/v PVA and vortexed for 20 seconds. The resulting emulsion was sonicated 1.5 min and the solvents were evaporated under gentle magnetic stirring.

#### Drug encapsulation in “green” pCD NPs

Encapsulation of ETH and Booster in pCD NPs was carried on without using any organic solvent, by mixing for at least 4 hours the NP suspensions with the drug powders.

### Nanoparticle characterization

#### Size measurement by PCS and Zeta Potential measurement

The average hydrodynamic diameter of the NPs was determined at 25 °C with an equilibration time of 60 s using a Malvern Zetasizer® (Nano ZS90, Malvern Instruments S.A., Worcestershire, UK). Dilutions with injectable water were done according to ISO 22412 and experiments were performed in triplicate. Mean diameters were reported as Z Average (nm) ± SE (Standard Error - with a PdI lower than 0.1) or as number mean diameter (nm) ± SD (Standard Deviation). The NPs mean diameters were monitored up to three months of storage at 25 °C. The NPs ZP was measured in KCl 1 mM by Malvern Zetasizer^©^.

#### Size measurement by NTA and concentration quantification

The mean hydrodynamic diameter and concentration of the NPs were measured at 25 °C by NTA (NanoSight LM10, Malvern Instruments S.A., Worcestershire, UK). NPs prepared by nanoprecipitation were diluted 2,000 times and the ones made by nanoemulsion 10,000 times. Each sample was measured 5 times for 60 seconds. Results were reported as mean diameter ± SD.

#### Cryo-TEM

5 µl of NPs suspensions were deposited onto a 200 mesh copper grid and flash-frozen in liquid ethane cooled down at liquid nitrogen temperature. Cryo-TEM images were acquired on a JEOL 2200FS energy-filtered (20 eV) field emission gun electron microscope operating at 200 kV. Several thousands of images were automatically acquired for each NPs formulation to obtain representative results.

### Drug(s) dosage

To determine the amounts of both drugs effectively incorporated in the PLA/PLGA NPs, the suspensions of drug-loaded NPs were centrifuged at 17,000 g for 15 minutes. Aliquots of the supernatants were withdrawn to assess the quantity of non-encapsulated drug (indirect estimation). The NPs pellet was dissolved in DMSO and dosed (direct estimation of the encapsulated drug). In the case of drug-loaded pCD NPs, excess non-encapsulated drug was removed by centrifugation, followed by NP dilution in DMSO and/or acetonitrile/water.

All the samples were dosed by liquid chromatography-mass spectrometry (LC-MS-MS) and reverse-phase HPLC (RP-HPLC) (For details see S.I. – Section III).

The DL can be defined as the mass fraction of a NP that is composed of drug, while the EE can be considered as the fraction of drug effectively encapsulated into the NPs compared with the amount that was used to prepare the NPs^20,54^. The DL and EE were calculated as shown in equation 1 and 2, respectively.

1 $$DL\,( \% )=\frac{(mg\,of\,encapsulated\,dru{g}^{\ast })}{(mg\,of\,polymer)}\times 100$$

2 $$EE\,( \% )=\frac{(mg\,of\,encapsulated\,dru{g}^{\ast })}{(mg\,of\,drug\,initially\,added\,to\,the\,formulation)}\times 100$$

[*The amount of encapsulated drug has been calculated with a direct or indirect estimation, depending on the sample fraction considered].

### Bacteria and macrophages

*M*. *tuberculosis* H37Rv strain (ATCC-27294) constitutively expressing the green fluorescent protein (H37Rv-GFP) was used as a reporter for the replication assay. Bacteria were cultured at 37 °C for 16 days in complete 7H9 medium containing 0.5% glycerol (50405, Euromedex), 10% Middlebrook oleic acid-albumin-dextrose-catalase (OADC, 211886, Becton Dickinson), 0.05% Tween 80 (2002A, Euromedex) and 50 µg/mL hygromycin B (10687010, Invitrogen). In the day of the experiment, *M*. *tuberculosis* were washed with D-PBS Ca- Mg- (14190169, LifeTechnologies) 3 times with centrifugation at 5,000 RPM for 5 minutes and centrifuged at 700 RPM for 2 minutes to remove clumped bacteria. Bacteria were resuspended in RPMI-1640 + glutamax (61870044, LifeTechnologies) containing 10% heat inactivated fetal bovine serum (FBS) (10270106, Gibco) and titrated by measuring the optical density at 600 nm.

Mouse macrophage RAW 264.7 (ATCC # TIB-71) were maintained at 37 °C in RPMI-1640 + glutamax containing 10% FBS and were passed 3 times per week and used before passage number 7. Macrophages were harvested by using Versene (15040033, LifeTechnologies).

### Assay plate preparation

ETH, BDM41906 and INH were diluted in DMSO (34943, Sigma-Aldrich) to 10 mg/mL and were dispensed in Echo-qualified 384-well low dead volume source plates (Labcyte). Echo 550 Series Liquid Handler (Labcyte) was used to transfer precise volumes between 5 and 500 nL from the Echo-qualified plate to the 384-well clear-bottom polystyrene assay plates (781091, Greiner Bio-One) by using sound waves. All the samples were backfilled with DMSO until 500 nL. Prior to addition of cells, 4.5 µL of cell medium were dispensed in the Echo-dispensed wells.

NPs loaded with ETH or with the [ETH:Booster] pair were diluted in water to 0.2 mg/mL of ETH. 2-fold serial dilutions of the mother solution in a final volume of 100 µL were performed in sterile MilliQ water in a 384 deep well “diamond plate” (P-384-120SQ-C-S, Axygen) in order to obtain a dose-response curve. Posteriorly, 5 µL of the NPs were dispensed in the 384-well assay plates.

### *M*. *tuberculosis* replication phenotypic assays

Bacteria were diluted at 2 × 10^6^ bacteria/mL using complete 7H9 medium and 45 µL/well of bacterial suspension were added in 384-well assay plates. After 5 days incubation at 37 °C, 5% CO_2_, extracellular plates were read using a fluorescence reader (Victor X3, Perkin Elmer) at excitation/emission of 485/535 nm for 0.1 seconds/well with a small emission aperture and CW-lamp energy of 50,000. The read-out, relative fluorescence units (RFU), versus the ETH concentration was then plotted using GraphPad Prism 5.0 software and the concentration required to inhibit 50% of the bacterial replication (IC_50_) was calculated by nonlinear regression analysis using the equation for a sigmoidal dose-response curve with variable slope.

For intracellular assay, bacteria were mixed with RAW 264.7 macrophages to prepare a suspension at 5 × 10^5^ cells/mL and 1 × 10^6^ bacteria/mL (multiplicity of infection 2) in RPMI-1640 + glutamax containing 10% FBS. After 2 hours of infection at 37 °C with shaking (120 RPM), infected cells were washed with RPMI-1640 + glutamax containing 10% FBS by centrifugation at 1,100 RPM for 5 minutes. The remaining extracellular bacilli from the infected cell suspension were killed by a 1 hour 50 μg/mL amikacin (A2324-5G, Sigma) treatment and then washed twice with RPMI-1640 + glutamax containing 10% FBS. Finally, 45 µL/well of cellular suspension was added in the 384-well assay plate and incubated during 5 days at 37 °C, 5% CO_2_. Macrophages were then stained with 5 µM Syto 60 (S11342, Molecular probes) dye for 1 hour, followed by plate sealing, imaging acquisition and data analysis.

Confocal images were recorded on an automated fluorescent ultra-high-throughput microscope Opera (Perkin Elmer) (For details see S.I. – Section III). A series of 6 pictures at the center of each well were taken and each image was then analysed using Columbus system version 2.5.1 as previously described^43^ to extract the intracellular bacterial area and the number of cells. The intracellular bacterial area was normalized with the negative control DMSO (0% inhibition) and the positive control INH at a concentration of 1 µg/mL (100% inhibition) by converting it into a percentage of bacterial replication inhibition (% inhibition). % inhibition was calculated as shown in the equation 3:

3 $$ \% \,\,inhibition=(1-\,\frac{{\rm{Test}}\,{\rm{bacterial}}\,{\rm{area}}-{\rm{INH}}\,{\rm{bacterial}}\,{\rm{area}}}{{\rm{DMSO}}\,{\rm{bacterial}}\,{\rm{area}}-{\rm{INH}}\,{\rm{bacterial}}\,{\rm{area}}})\times 100$$

For each compound, a plot of % inhibition versus the ETH concentration was determined with GraphPad Prism 5.0 software and the IC_50_ was calculated in the same way as in the extracellular assay.

### *In vivo* experiments

6-week old Balb/C female mice were purchased from Janvier (Le Genest-Saint-Isle, France) and were maintained in the animal house facility of the Pasteur Institute of Lille, France (Agreement B59-350009). The project received ethical approval by French Committee on Animal Experimentation and the Ministry of Education and Research (00579.01 approved on December 2^nd^ 2015) and all experiments were performed in accordance with relevant guidelines and regulations.

#### Lung histology

Infected or uninfected 8-week-old mice were divided in groups and endotracheally administered with water (vehicle) or pCD NPs (Figs 5a and 6).

At the determined end-point mice were euthanized, lungs were harvested and soaked in 4% formaldehyde (10% formalin solution, neutral buffered, HT501128, Sigma-Aldrich) for 24 hours, before being embedded in paraffin. Tissues were sliced with microtome and 5 µm sections were stained with Hematoxylin-Eosin (H-E) for light microscopy examination for anatomopathology.

#### Flow cytometry

Harvested lungs were cut into small pieces and incubated for 1 hour at 37 °C with a mix of DNAse I (100 μg/ml, Sigma-Aldrich) and collagenase (1.6 mg/ml, Roche) 400 U/ml. Lung cells were washed and filtered before being incubated with saturating doses of purified 2.4G2 (anti-mouse Fc receptor, ATCC) in 200 μl PBS 0.2% BSA 0.02% NaN3 (FACS buffer) for 20 minutes at 4 °C to prevent antibody binding on the Fc receptor. Various fluorescent mAb combinations in FACS buffer were used to stain 3–5 × 10^6^ cells. Acquisitions were done on FACScanto II cytofluorometer (Becton Dickinson) with the following mAbs from BD Biosciences: Fluorescein (FITC)-coupled HL3 (anti-CD11c), FITC-coupled 145-2C11 (anti-CD3), APC-coupled RB6-8C5 (anti-GR1), phycoérythrine (PE)-coupled RM4-5 (anti-CD4), PE-coupled E50-2440 (anti-SIGLEC-F), APC-coupled BM8 (anti-F4/80). APC-eF780-coupled M1/70 (antiCD11b) were purchased from eBiosciences and fixable viability dye eFluor 506 (eBiosciences) was used to gate viable cells.

#### Efficacy studies

8-week-old mice (4 mice *per* group) were inoculated with *M*. *tuberculosis* H37Rv via the intranasal route to reach 10^6^ bacteria in the lungs at day 0. NPs or INH were administered to mice using a Microsprayer® (MicroSprayer® Aerosolizer – Model IA-1C-M and FMJ-250 High Pressure Syringe, Penn Century Inc., Wyndmoor, PA). For the 1-week treatment and the 2-week treatment, administrations were performed on day 7, 9, 11 and on day 7, 9, 11, 14, 16, 18 respectively. The body weight of mice was monitored after each treatment (Fig. 5b). To assess the reproducibility of NPs administration through the MicroSprayer®, the delivered doses of NP suspensions were collected in glass vials after each spray and were accurately weighed. Then the amount of the delivered drug was quantified by HPLC as already described.

The protocol to administer the NPs in mice was adapted from a previously reported one^55^. Briefly, mice were placed in isoflurane chamber (Aerrane®, Baxter SAS, France). Each mouse was placed on the back on a platform (Mouse Intubation Platform – Model MIP, Penn Century Inc., Wyndmoor, PA) with isoflurane mask and hanging on its teeth. The tongue was pulled out by a tweezer and a laryngoscope (Small Animal Laryngoscope for mouse – Model LS-2-M, Penn Century Inc., Wyndmoor, PA) was used to see the trachea and to enable the aerosolization of 50 µL of NP suspensions inside the lungs.

At day 14 or 21, mice were euthanized and lungs were homogenized with MM300 bead beater (Retsch) and eight ten-fold serial dilutions were plated onto 7H11 agar plates supplemented with 10% OADC. CFUs were determined after a three-weeks growth. Represented data are mean values ± standard deviation of one representative experiment of three independent experiments. Results for each independent experiment were summarized in Supplementary Table S2. Statistics were performed using Student’s t-test and one-way ANOVA analysis. Same p-values for *in vivo* experiments were obtained with the two tests. *p < 0.1, **p < 0.01,***p < 0.001.

## Electronic supplementary material

Supplementary information

Supplementary Video S1

## References

[1] Global tuberculosis report 2016 (WHO 2016).

[2] O’Garra A (2013). The immune response in tuberculosis. Annu Rev Immunol.

[3] Gandhi NR (2010). Multidrug-resistant and extensively drug-resistant tuberculosis: a threat to global control of tuberculosis. Lancet.

[4] Ethionamide. *Tuberculosis* **88**, 106–108, doi:10.1016/s1472-9792(08)70009-x.

[5] Zhu M (2002). Population pharmacokinetics of ethionamide in patients with tuberculosis. Tuberculosis (Edinb).

[6] Baulard AR (2000). Activation of the pro-drug ethionamide is regulated in mycobacteria. Journal of Biological Chemistry.

[7] Vannelli TA, Dykman A, de Montellano PRO (2002). The antituberculosis drug ethionamide is activated by a flavoprotein monooxygenase. J. Biol. Chem..

[8] Willand N (2009). Synthetic EthR inhibitors boost antituberculous activity of ethionamide. Nat. Med..

[9] Mehanna MM, Mohyeldin SM, Elgindy NA (2014). Respirable nanocarriers as a promising strategy for antitubercular drug delivery. J Control Release.

[10] Pham DD, Fattal E, Tsapis N (2015). Pulmonary drug delivery systems for tuberculosis treatment. Int J Pharm.

[11] Hickey AJ, Durham PG, Dharmadhikari A, Nardell EA (2015). Inhaled drug treatment for tuberculosis: Past progress and future prospects. J Control Release.

[12] Pandey R (2003). Poly (DL-lactide-co-glycolide) nanoparticle-based inhalable sustained drug delivery system for experimental tuberculosis. J Antimicrob Chemother.

[13] Ahmad Z, Sharma S, Khuller GK (2005). Inhalable alginate nanoparticles as antitubercular drug carriers against experimental tuberculosis. Int J Antimicrob Agents.

[14] Bivas-Benita M, Ottenhoff TH, Junginger HE, Borchard G (2005). Pulmonary DNA vaccination: concepts, possibilities and perspectives. J Control Release.

[15] Costa A (2016). The formulation of nanomedicines for treating tuberculosis. Adv Drug Deliv Rev.

[16] Flipo M (2011). Ethionamide boosters. 2. Combining bioisosteric replacement and structure-based drug design to solve pharmacokinetic issues in a series of potent 1, 2, 4-oxadiazole EthR inhibitors. J. Med. Chem..

[17] Lopes E, Pohlmann AR, Bassani V, Guterres SS (2000). Polymeric colloidal systems containing ethionamide: preparation and physico-chemical characterization. Pharmazie.

[18] Bhanushali CJ, Zidan AS, Rahman Z, Habib MJ (2013). Ion-pair chromatography for simultaneous analysis of ethionamide and pyrazinamide from their porous microparticles. Aaps Pharmscitech.

[19] Vale N (2012). New times, new trends for ethionamide: *In vitro* evaluation of drug-loaded thermally carbonized porous silicon microparticles. Eur J Pharm Biopharm.

[20] Kumar G (2011). *In vitro* physicochemical characterization and short term *in vivo* tolerability study of ethionamide loaded PLGA nanoparticles: potentially effective agent for multidrug resistant tuberculosis. J Microencapsul.

[21] Davis ME, Brewster ME (2004). Cyclodextrin-based pharmaceutics: past, present and future. Nat Rev Drug Discov.

[22] Committee for Human Medicinal Products (CHMP) Background review for cyclodextrins used as excipients. *European Medecines Agency* (2014).

[23] Stella VJ, He Q (2008). Cyclodextrins. Toxicol Pathol.

[24] Zuckerman JE (2014). Correlating animal and human phase Ia/Ib clinical data with CALAA-01, a targeted, polymer-based nanoparticle containing siRNA. Proc Natl Acad Sci USA.

[25] Daoud-Mahammed S (2009). Cyclodextrin and polysaccharide-based nanogels: entrapment of two hydrophobic molecules, benzophenone and tamoxifen. Biomacromolecules.

[26] Daoud-Mahammed S (2008). Self-assembling cyclodextrin based hydrogels for the sustained delivery of hydrophobic drugs. J Biomed Mater Res A.

[27] Davis ME (2010). Evidence of RNAi in humans from systemically administered siRNA via targeted nanoparticles. Nature.

[28] Gref R (2006). New self-assembled nanogels based on host-guest interactions: characterization and drug loading. J Control Release.

[29] Christophe T, Ewann F, Jeon HK, Cechetto J, Brodin P (2010). High-content imaging of Mycobacterium tuberculosis-infected macrophages: an *in vitro* model for tuberculosis drug discovery. Future Med Chem.

[30] Christophe T (2009). High content screening identifies decaprenyl-phosphoribose 2′ epimerase as a target for intracellular antimycobacterial inhibitors. PLoS Pathog.

[31] Queval CJ (2016). STAT3 Represses Nitric Oxide Synthesis in Human Macrophages upon Mycobacterium tuberculosis Infection. Sci Rep.

[32] Sorrentino F (2015). Development of an Intracellular Screen for New Compounds Able To Inhibit Mycobacterium tuberculosis Growth in Human Macrophages. Antimicrob Agents Chemother.

[33] Stanley SA (2014). Identification of host-targeted small molecules that restrict intracellular Mycobacterium tuberculosis growth. PLoS Pathog.

[34] Costa-Gouveia, J., Ainsa, J. A., Brodin, P. & Lucia, A. How can nanoparticles contribute to antituberculosis therapy? *Drug Discov Today* (2017). 10.1016/j.drudis.2017.01.011 28137645

[35] Layre AM (2006). Busulfan loading into poly(alkyl cyanoacrylate) nanoparticles: physico-chemistry and molecular modeling. J Biomed Mater Res B Appl Biomater.

[36] Bouligand J (2007). Busulphan-loaded long-circulating nanospheres, a very attractive challenge for both galenists and pharmacologists. J Microencapsul.

[37] Filipe V (2011). Fluorescence single particle tracking for the characterization of submicron protein aggregates in biological fluids and complex formulations. Pharm Res.

[38] Filipe V, Hawe A, Jiskoot W (2010). Critical evaluation of Nanoparticle Tracking Analysis (NTA) by NanoSight for the measurement of nanoparticles and protein aggregates. Pharm Res.

[39] Li Y, Lubchenko V, Vekilov PG (2011). The use of dynamic light scattering and brownian microscopy to characterize protein aggregation. Rev Sci Instrum.

[40] Zambaux MF, Bonneaux F, Gref R, Dellacherie E, Vigneron C (1999). Preparation and characterization of protein C-loaded PLA nanoparticles. J Control Release.

[41] Zambaux MF (1998). Influence of experimental parameters on the characteristics of poly(lactic acid) nanoparticles prepared by a double emulsion method. J Control Release.

[42] Kumar G, Shafiq N, Malhotra S (2012). Drug-loaded PLGA nanoparticles for oral administration: fundamental issues and challenges ahead. Crit Rev Ther Drug Carrier Syst.

[43] Queval, C. J. *et al*. A microscopic phenotypic assay for the quantification of intracellular mycobacteria adapted for high-throughput/high-content screening. *J Vis Exp* e51114, doi:10.3791/51114 (2014). 10.3791/51114 PMC4089478 24473237

[44] Guillon A (2012). Pulmonary delivery of dry powders to rats: tolerability limits of an intra-tracheal administration model. Int J Pharm.

[45] Wong BA (2007). Inhalation exposure systems: design, methods and operation. Toxicol Pathol.

[46] Maillet A (2011). The airways, a novel route for delivering monoclonal antibodies to treat lung tumors. Pharm Res.

[47] Chandenier J (2009). The utility of a nebulised intra-tracheal rat model of invasive pulmonary aspergillosis. Mycoses.

[48] Gagnadoux F (2005). Aerosol delivery of chemotherapy in an orthotopic model of lung cancer. The European respiratory journal.

[49] Aragao-Santiago L (2016). Compared *in vivo* toxicity in mice of lung delivered biodegradable and non-biodegradable nanoparticles. Nanotoxicology.

[50] Cabral Marques HM, Hadgraft J, Kellaway IW, Taylor G (1991). Studies of cyclodextrin inclusion complexes. IV. The pulmonary absorption of salbutamol from a complex with 2-hydroxypropyl-β-cyclodextrin in rabbits. Int J Pharm.

[51] Evrard B (2004). Cyclodextrins as a potential carrier in drug nebulization. J Control Release.

[52] Othman M (2011). A comprehensive study of the spontaneous formation of nanoassemblies in water by a “lock-and-key” interaction between two associative polymers. J Colloid Interface Sci.

[53] Bilati U, Allémann E, Doelker E (2005). Nanoprecipitation versus emulsion-based techniques for the encapsulation of proteins into biodegradable nanoparticles and process-related stability issues. Aaps Pharmscitech.

[54] Ankrum JA (2014). Engineering cells with intracellular agent–loaded microparticles to control cell phenotype. Nat. Protocols.

[55] Bivas-Benita M, Zwier R, Junginger HE, Borchard G (2005). Non-invasive pulmonary aerosol delivery in mice by the endotracheal route. Eur J Pharm Biopharm.

