# Publisher Correction: Combination therapy for tuberculosis treatment: pulmonary administration of ethionamide and booster co-loaded nanoparticles

**DOI:** 10.1038/s41598-018-25177-2

**Published:** 2018-05-10

**Authors:** Joana Costa-Gouveia, Elisabetta Pancani, Samuel Jouny, Arnaud Machelart, Vincent Delorme, Giuseppina Salzano, Raffaella Iantomasi, Catherine Piveteau, Christophe J. Queval, Ok-Ryul Song, Marion Flipo, Benoit Deprez, Jean-Paul Saint-André, José Hureaux, Laleh Majlessi, Nicolas Willand, Alain Baulard, Priscille Brodin, Ruxandra Gref

**Affiliations:** 1 Univ. Lille, CNRS, INSERM, CHU Lille, Institut Pasteur de Lille, U1019 - UMR 8204 - CIIL - Center for Infection and Immunity of Lille, F-59000 Lille, France; 2 University of Paris-Sud, University Paris-Saclay, CNRS, UMR 8214 - Institute for Molecular Sciences of Orsay (ISMO), 91405 Orsay, France; 3 Univ. Lille, INSERM, Institut Pasteur de Lille, U1177 - Drugs and Molecules for living Systems, F-59000 Lille, France; 4 University Hospital Center of Angers, 49000 Angers, France; 5 Pathogénomique Mycobactérienne Intégrée, Département de Génomes et Génétique, Institut Pasteur, Paris, France

Correction to: *Scientific Reports* 10.1038/s41598-017-05453-3, published online 14 July 2017

In the original version of this Article, the Author information section was incomplete. Alain Baulard was omitted as having jointly supervised this work. This has now been corrected in the HTML and PDF versions of this Article.

