# Supplementary material for: Combination therapy for tuberculosis treatment: pulmonary administration of ethionamide and booster co-loaded nanoparticles
Source: Sci Rep. 2017 Jul 14;7:5390. doi: 10.1038/s41598-017-05453-3 (PMC5511234; doi:10.1038/s41598-017-05453-3)
Supplement: Supplementary file 1 — Supplementary information [file 41598_2017_5453_MOESM1_ESM.pdf]

Combination therapy for tuberculosis treatment: pulmonary administration of ethionamide and booster co-loaded nanoparticles

Joana Costa-Gouveia[1#], Elisabetta Pancani[2#], Samuel Jouny[1], Arnaud Machelart[1], Vincent Delorme[1], Giuseppina Salzano[2], Raffaella Iantomasi[1], Catherine Piveteau[3], Christophe J Queval[1], Ok-Ryul Song[1], Marion Flipo[3], Benoit Deprez[3&], Jean-Paul Saint André[4], José Hureaux[4&], Laleh Majlessi[5&], Nicolas Willand[3&], Alain Baulard[1&], Priscille Brodin[1*&], Ruxandra Gref[2*&]

[1]Univ. Lille, CNRS, INSERM, CHU Lille, Institut Pasteur de Lille, U1019 - UMR 8204 - CIIL - Center for Infection and Immunity of Lille, F-59000 Lille, France

[2]University of Paris Sud, University Paris-Saclay, CNRS, UMR 8214 - Institute for Molecular Sciences of Orsay (ISMO), 91405 Orsay, France

[3] Univ. Lille, INSERM, Institut Pasteur de Lille, U1177 - Drugs and Molecules for living Systems, F-59000 Lille, France

[4]University Hospital Center of Angers, 49000 Angers, France

[5]Pathogénomique Mycobactérienne Intégrée, Département de Génomes et Génétique, Institut Pasteur, Paris, France

[#]equally contributing first authors

*corresponding authors: Gref, Ruxandra, (ruxandra.gref@u-psud.fr), Brodin, Priscille (priscille.brodin@inserm.fr)
[&]co-senior authors

Supplementary Information

Section I - Nanoparticles development

Section II - Phenotypic extracellular and intracellular assays and *in vivo* results

Section III - Detailed materials and methods

Section I - Nanoparticles development

PLA/PLGA NPs – Nanoprecipitation method

Several PLA and PLGA polymers were employed to prepare the nanoparticles by nanoprecipitation (P1, P2, P3, P4, see materials and method section). In all cases the formulated NPs were remarkably stable upon storage up to 5 months, with less than 5% size variation. The size variation over a period of 1 month is shown in the Supplementary Fig. S1.

Interestingly no difference in the size evolution of NPs was shown between the formulation stored at room temperature or in the refrigerator (4°C).

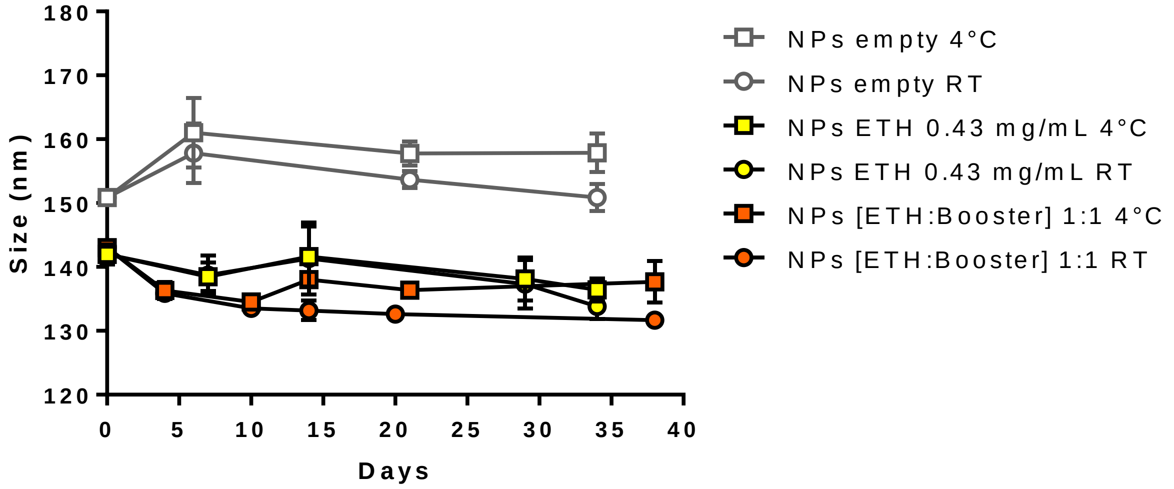

Supplementary Figure S1 – Stability of nanoparticles obtained by nanoprecipitation method over a period of 1 month (measured by DLS). The graph shows size stability of nanoparticles (charged or not) over 1 month. RT: storage at room temperature, 4°C: storage in the refrigerator.

The first attempt of formulation has been performed using P1 (1.43 mg/mL polymer in suspension). The encapsulation of Ethionamide (ETH) alone was studied by trying to encapsulate increasing concentrations of this drug (from 0.07 to 0.71 mg/mL). The results are shown in the Supplementary Fig. S2.

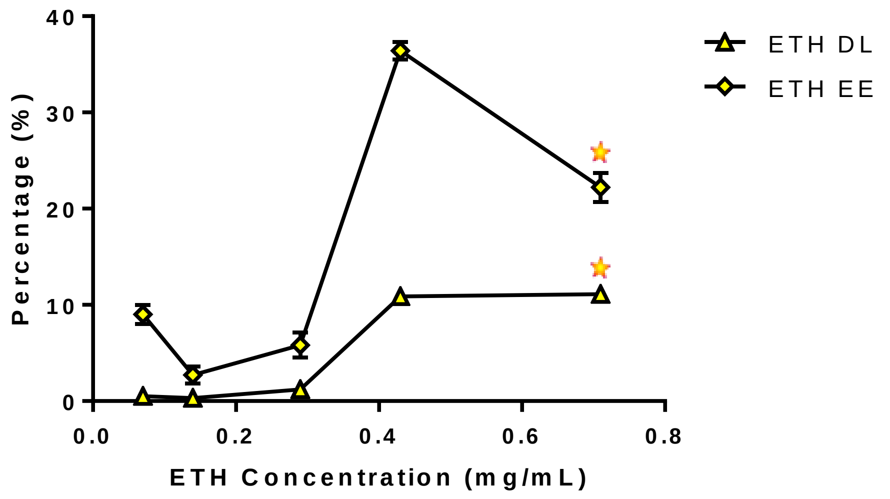

Supplementary Figure S2 – Effect of increasing ETH concentration on EE% and DL% of nanoparticles formulated by nanoprecipitation with P1. The yellow star (★) represents the formation of ETH crystals few days after nanoparticles preparation (ETH concentration of 0.71 mg/mL).

Formulations up to an ETH concentration of 0.43 mg/mL were completely stable. In these conditions the highest achieved drug loading (DL) and encapsulation efficiency (EE) were 11 wt % and 36 wt %, respectively. Unfortunately, raising the amount of ETH to 0.71 mg/mL in the attempt to further increase DL and EE, the DL resulted unchanged and the encapsulation efficiencies decreased to 22 % (Supplementary Fig. S2). Moreover, increasing ETH amounts in the NP preparation procedure lead to the formation of drug crystals which progressively grew in the suspension media until being seen with the naked eye, few days after NP preparation (Supplementary Fig. S3).

Supplementary Fig. S3 shows the decreasing of DL and EE (P1 and ETH concentration of 0.71 mg/mL) during the first 8 days after NPs formulation. The formation of crystals was observed after 5 days and corresponds to a further decrease in NPs DL and EE.

ETH crystallisation in P1 NPs was avoided only at ETH concentrations below 0.71 mg/mL, which is insufficient for *in vivo* applications. Attempts to concentrate the NPs suspensions failed because of a strong aggregation in the absence of protective surfactants.

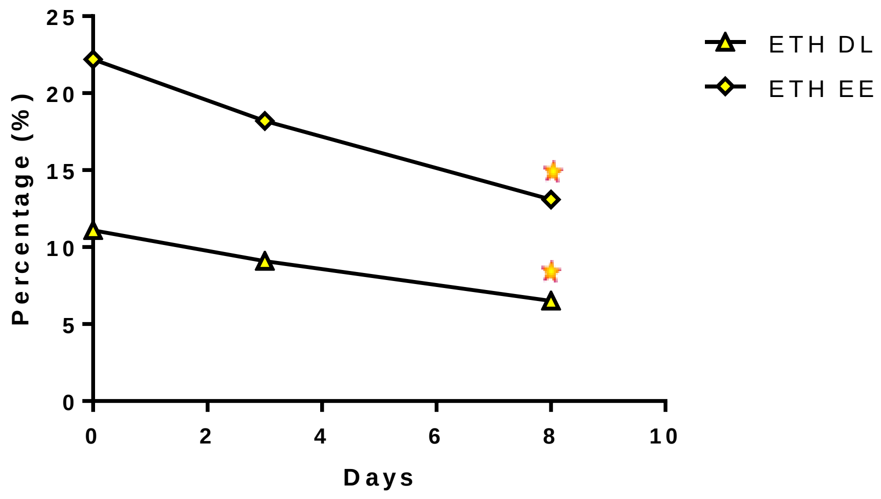

Supplementary Figure S3 - DL% and EE% decrease during storage. DL and EE decreased during first days of storage leading to the formation of crystals at the bottom of the flask (represented by the yellow star ⭐ in the figure) and to a decrease of both DL and EE.

In an additional attempt to increase both DL and EE, a series of PLA and PLGA copolymers were used, with different molecular weights and compositions (P2, P3 and P4). In all cases, aggregation or drug crystallization occurred. Better results in terms of DL and EE (24 wt % and 34 wt %) were obtained with P3 and an ETH concentration of 1 mg/mL. However, crystallisation occurred after 5 days as for the other polymers. Moreover, co-encapsulating Booster did not improve ETH loading, as it was found that Booster had stronger affinity for the polymeric NP than ETH. As an example, at the ratios [ETH:Booster] [1:0.1] and [1:1], ETH DL were lower than 2 wt %. Interestingly Booster DL and EE during coencapsulation were significantly higher than ETH ones (9 % and 29 % wt respectively, for ratio [1:1]) showing a higher affinity of the Booster for the polymeric matrix.

PLA NPs – Nanoemulsion method

To address the challenge of coencapsulating ETH and Booster, a second method based on the formation of an emulsion, stabilized with polyvinyl alcohol (PVA), a surfactant approved for pulmonary administration, was set up using P4 (see material and methods section).

An extensive study on mean diameter of PLA NPs obtained by nanoemulsion has been performed using the 3 independent methods: PCS, NTA and cryo-TEM (see main text). The

results show the importance of the use of different techniques to truly characterise nanoparticulate systems. Results are reported in Supplementary Table S1. It's interesting to point out that no significant difference in the diameter of NPs has been found between empty PLA NPs and drug-loaded PLA NPs.

Supplementary Table S1 - Main characteristics of empty and drug-loaded NPs made of P4 by the nanoemulsion method. NP mean hydrodynamic diameters were determined by PCS and NTA. Nanoparticles dry diameter was determined by cryo-TEM and nanoparticle concentration was obtained by NTA. Concentration results given were obtained after 10,000 times dilution of each sample as prescribed to fit the instrument requirements and to allow the particle to move freely according to their Brownian motions.

Formulation	Mean diameter					Concentration
	**D PCS** Z Average (nm ± SE)	**PdI PCS**	**D PCS** Number (nm ± SE)	**D NTA** (nm± SD)	**Cryo-TEM**	**NTA** (particle/mL)
**NPs**	254 ± 4	0.054	220 ± 10	176 ± 45	110 ± 50	$1.01x10^9$
**NPs [ETH]**	267 ± 3	0.074	226 ± 10	176 ± 48	100 ± 40	$0.88x10^9$
**NPs [ETH:Booster] [1:0.1]**	258 ± 3	0.058	223 ± 10	180 ± 51	100 ± 50	$1.03x10^9$
**NPs [ETH:Booster] [1:1]**	274 ± 4	0.090	235 ± 11	178 ± 52	100 ± 50	$0.89x10^9$
**NPs [Booster]**	277 ± 5	0.075	238 ± 11	171 ± 49	120 ± 55	$0.91x10^9$

NPs formulated with the nanoprecipitation method were remarkably stable upon storage more than 5 months, with less than 10 % size variation and a PdI still lower than 0.1. The size variation over a period of 1 month is shown in the Supplementary Fig. S4

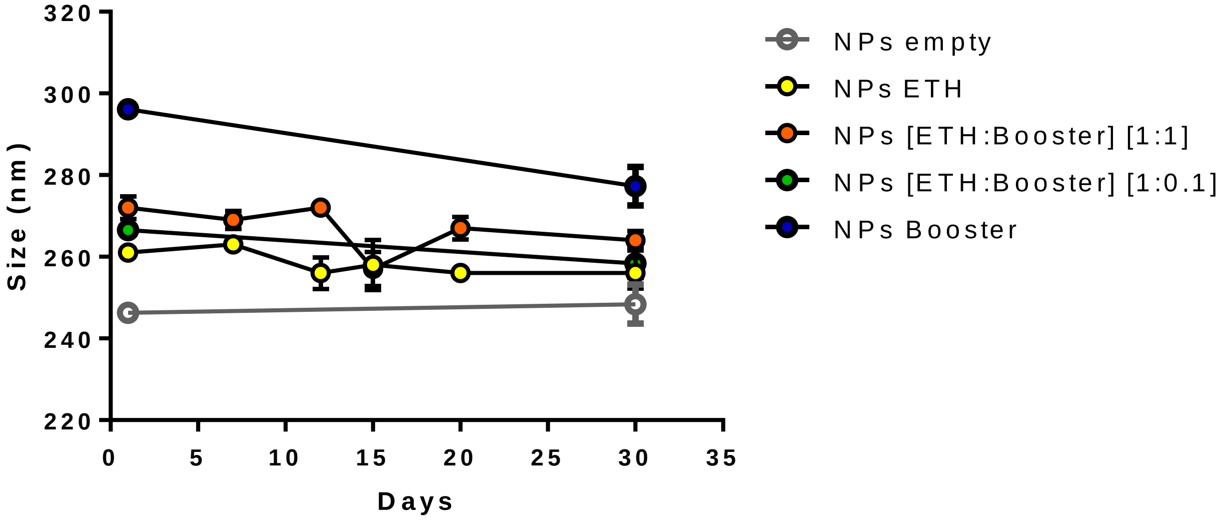

Supplementary Figure S4 – Stability of NPs obtained by nanoemulsion over a period of 1 month. No crystallization was observed during storage and no significant size or polydispersity changes (DLS) were found.

pCD NPs – Stability and release properties

The drug-loaded particles were stable upon storage and drugs were released only upon dilution of the NPs suspensions. Indeed, the drug molecules inside pCD NPs are in equilibrium with the ones in the suspension medium. Both ETH and Booster, poorly soluble in aqueous solution, strongly bound to pCD NPs. For instance, if the NPs are diluted ten times, one can calculate that around 90% of Booster will still be associated to the NPs.
The mechanism of drug release from pCD NPs, based on a partition mechanisms, has been thoroughly described in our previous publications [1-3].

Section II - Phenotypic extracellular and intracellular assays and in vivo results

To investigate the efficacy of drug-loaded NPs against *M. tuberculosis,* we used two phenotypic assays that are disease-relevant and amenable to high-throughput. The first one is a simple assay relying on the monitoring of fluorescence from a strain of *M. tuberculosis* that expresses the green fluorescence protein using a microplate photometer. The second is an image-based model that allows the multi-parametric quantification of *M. tuberculosis* replication inside its favourite niche, the macrophage. The dedicated protocol used for image analysis with the Columbus software is given in Supplementary S5 below.

**Input Image**	**Stack Processing :** Individual<br>**Flatfield Correction :** None		
**Find Nuclei**	**Channel :** Exp1Cam3<br>**ROI :** None	**Method :** B<br>Common Threshold : 0.5<br>Area : > 15 μm^2<br>Split Factor : 5<br>Individual Threshold : 0.55<br>Contrast : > 0.2	Output Population : Nuclei
**Select Population (5)**	**Population :** Nuclei	**Method :** Common Filters<br>Remove Border Objects<br>Region : Nucleus	Output Population : Nuclei 2
**Calculate Intensity Properties (2)**	**Channel :** Exp1Cam3<br>**Population :** Nuclei 2<br>**Region :** Nucleus	**Method :** Standard<br>Mean	Output Properties : Intensity Nucleus Exp1Cam3
**Select Population (3)**	**Population :** Nuclei 2	**Method :** Filter by Property<br>Intensity Nucleus Exp1Cam3 Mean : > 200	Output Population : Nuclei 2 Selected
**Find Cytoplasm**	**Channel :** Exp1Cam3<br>**Nuclei :** Nuclei 2 Selected	**Method :** A<br>Individual Threshold : 0.15	
**Find Spots**	**Channel :** Exp1Cam2<br>**ROI :** Nuclei 2 Selected<br>**Region :** Cell	**Method :** B<br>Detection Sensitivity : 0.55<br>Splitting Coefficient : 1<br>Calculate Spot Properties	Output Population : Spots
**Calculate Intensity Properties**	**Channel :** Exp1Cam2<br>**Population :** Spots<br>**Region :** Spot	**Method :** Standard<br>Mean	Output Properties : Intensity Spot Exp1Cam2
**Select Population**	**Population :** Spots	**Method :** Filter by Property<br>Intensity Spot Exp1Cam2 Mean : > 150<br>Spot Contrast : > 0.2<br>Boolean Operations : F1 and F2	Output Population : Bacteria
**Calculate Morphology Properties (2)**	**Population :** Bacteria<br>**Region :** Spot	**Method :** Standard<br>Area	Output Properties : Bacteria
**Define Results**	**Method :** List of Outputs<br>**Population : Nuclei 2 Selected**<br>Number of Objects<br>**Population : Bacteria**<br>Bacteria Area [μm^2] : Sum		

Supplementary Figure S5- Columbus script used for image analysis. Two colour-images were analysed. The red recorded on the channel "Exp1Cam3" was used for the nucleus and cytoplasm detection. The green recorded on the channel "Exp1Cam2" was used to detect the bacteria. ROI: Region of interest.

In Supplementary Fig. S6 we point out that the % of inhibition in the presence of empty PLA and empty pCD is negligible (close to zero) independently from the concentration of the NPs, both in the intra- and extracellular assay. These results show that the antibacterial effect observed is due to the presence of ETH and Booster released from the nanoparticles since the unloaded nanoparticles were not able to impair bacterial replication.

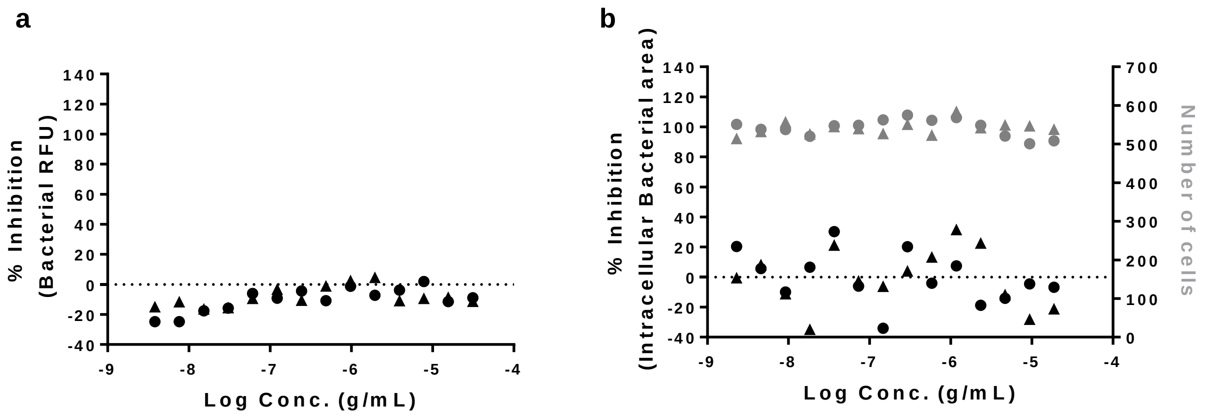

Supplementary Figure S6- Effect of empty NPs on intra- and extra-cellular assay. (a) % of inhibition of bacterial RFU obtained with empty PLA made by the nanoemulsion method (solid circle) and with empty pCD (solid triangle). **(b)** % of inhibition of intracellular bacterial area (black points) and number of cells (grey points) obtained with empty PLA made by the nanoemulsion method (solid circle) and with empty pCD (solid triangle).

In Supplementary Table S2 we present the results of 3 independent *in vivo* experiments after 2 weeks of treatment as described in the manuscript. In the 3 experiments we observed a significant decrease in the pulmonary bacterial load after treatment with ETH and [ETH:Booster] encapsulated in pCD NPs.

Supplementary Table S2- Comparison of 3 independent in vivo experiments.

Log10 CFU in lungs mean ± SD at 21 days post infection	**Control**	**pCD [ETH]**	**pCD [ETH:booster]**
Exp 1	**9.15 +/- 0,44**	**7.83 +/- 0.59 ***	**5.77 +/- 0.27 *****
Exp 2	**7.51 +/- 0.16**	**6.25 +/- 0.28 *****	**5.87 +/- 0.48 *****
Exp 3	**7.22 +/- 0.23**	**6.04 +/- 0.72 ***	**5.12 +/- 0.28 *****

*:p<0.1. ***:p<0.001

Section III - Detailed Materials and methods

Chemicals

PLGA 75:25 acid terminated (P1) (MW: 37-84 KDa, 10P002), PLGA 50:50 ester terminated (P2) (MW: 70-100 KDa, 10P016) and PLGA 50:50 acid terminated (P3) (MW: 5-20 KDa, 10P019) were kindly provided by PCAS (Expansorb, Aramon, France). PLA ester terminated (P4) (MW =10-18 kDa), PVA (87-90% hydrolyzed), DMSO-99.5%, ETH and amikacin were all purchased from Sigma-Aldrich. Injectable water was purchased from Cooper (Melun, France). Dichloromethane (DCM-Analar Normapur stabilized with about 0.002% of 2-methyl-2-butene) and MeOH were from Prolabo (Fontenay-sous-Bois, France). KCl (99%) was purchased from Alfa Aesar (Karlsruhe, Germany).

The Booster named BDM41906 (5,5,5-Trifluoro-1-[4-(3-thiazol-2-yl-1,2,4-oxadiazol-5-yl)piperidin-1-yl] pentan-1-one) was synthesized as previously described [4].

β-CD was kindly supplied by Roquette, Lestrem, France. pCD NPs of around 10 nm were produced as previously described [5,6] by crosslinking β-CD under strongly alkaline conditions with epichlorohydrin (EP). Briefly, 100 g of anhydrous β-CD was solubilized overnight in 160 mL of NaOH 33% w/w solution. Then, 81.52 g of EP was rapidly added and the reaction was stopped in the vicinity of the gelation point by addition of acetone. The β-CD NPs, recovered by ultrafiltration followed by freeze-drying, contained 70% w/w β-CD, as determined by ^{1}H NMR spectroscopy.

Nanoparticle preparation

Nanoprecipitation: encapsulation of ETH in PLA and PLGA NPs was carried out adapting a previously reported procedure [7]. Briefly, 0.4 mL of DMSO solution containing 10 mg polymer (PLA or PLGA) and ETH (0.5 to 7 mg) were poured drop by drop in 7 mL of injectable water under magnetic stirring. In the case of co-encapsulation, 3 mg (ratio 10:10) or 0.3 mg of Booster (ratio 10:1) were added to the DMSO solution.

Nano-emulsion: preparation of PLA NPs was carried out as previously reported by Kumar et al. with slight modifications [8]. Briefly, 10 mg of ETH were solubilized in 0.2 mL of MeOH and mixed to 1.5 mL of a dichloromethane (DCM) solution containing 20 mg of P4 in DCM.

For the co-encapsulation of the synergic drugs pair, 10 mg of Booster, in case of 10:10 ratio, or 1 mg of Booster, in case of 10:1 ratio, were solubilized in the solution of PLA in DCM. Control empty NPs were prepared in the same conditions, except that the drugs were not added.

The organic phase was poured into 4 mL of an aqueous phase (injectable water) containing 0.5% w/v PVA and emulsified 20 seconds (vortex). The mixture was then sonicated (Sonopuls HD 2070, BANDELIN electronic GmbH & Co, Berlin, Germany) at 20% power for 1.5 min, stopping the procedure each 30 seconds to avoid the overheating and for 30 additional seconds to 10%. The resulting emulsion was left uncapped under magnetic stirring, checking the weight of the flask to monitor the evaporation of the organic solvents, until complete evaporation of DMC and MeOH.

Drug encapsulation in "green" pCD NPs: encapsulation of ETH and Booster in pCD NPs was carried on without using any organic solvent, by mixing for at least 4 hours the NP suspensions with the drug powders.

Nanoparticle characterization

Size measurement by PCS and Zeta Potential measurement

The average hydrodynamic diameter of the NPs was determined at 25˚C, in triplicate, with an equilibration time of 60 s, using a Malvern Zetasizer® (Nano ZS90, Malvern Instruments S.A., Worcestershire, UK). For the measurement of method 1 formulations, Samples were analyzed without performing any dilution (method 1) and with 1:10 and 1:100 dilutions in injectable water (as prescribed by ISO 22412) in the case of method 2. No significant differences were noticed in the hydrodynamic diameter of the two dilutions. All autocorrelation functions have an intercept with amplitude equal to 0.9 or higher. Mean diameters were reported as Z Average (nm) ±SE (Standard Error - with a Polydispersity Index lower than 0.1) or as number mean diameter (nm) ±SD (Standard Deviation).

The stability of the NPs prepared by nanoprecipitation and nanoemulsion was assessed at RT (25°C-storage conditions). The NPs mean diameters were monitored up to three months of storage at 25°C via DLS size measurements (see Supplementary Fig. S1 and S5).

Also the Zeta Potential (ZP) of nanoparticles was measured in KCl 1Mm by Malvern Zetasizer©. Each sample was diluted 100 times and 1,000 times in KCl 1mM. Results are reported as ZP (mV) ± SD.

Size measurement by NTA and concentration quantification

Mean hydrodynamic diameter and concentration of the NPs were measured 5 times for 60 seconds at 25°C by NTA (NanoSight LM10, Malvern Instruments S.A., Worcestershire, UK, equipped with a 565 nm filter and a NanoSight syringe pump). NPs prepared by nanoprecipitation were diluted 2,000 times and the ones made by nanoemulsion 10000 times. Results are reported as mean diameter ± SD.

Cryogenic transmission electron microscopy (cryo-TEM)

5 µl of NPs suspensions (diluted 4 times in water) were deposited onto a 200 mesh holey copper grid (Ted Pella Inc.) and flash-frozen in liquid ethane cooled down at liquid nitrogen temperature. Cryo-TEM images were acquired on a JEOL 2200FS energy-filtered (20eV) field emission gun electron microscope operating at 200 kV using a Gatan ssCCD 2048 × 2048 pixels. Several thousands of images were automatically acquired for each NP formulation to obtain representative results.

Drug sample preparation for drug(s) dosage

To determine the amounts of ETH and Booster incorporated in PLGA NPs, 500 µL of NP suspensions were centrifuged at 17,000g for 15 minutes. Then, 200 µL of the supernatant were withdrawn to assess the quantity of non-encapsulated drug (indirect estimation). The NPs pellet was dissolved in 250 µL DMSO and dosed (direct estimation of the encapsulated drug). For the pCD NPs, aliquots of pCD NPs containing drug(s) were centrifuged at 5000 rpm for 10 min to remove unloaded drug(s). The supernatant containing the pCD NPs was then diluted with DMSO (1:1 volume ratio) and dosed by HPLC after further dilution in DMSO and/or with acetonitrile/water 45/55 v/v.

All the samples were dosed by liquid chromatography-mass spectrometry (LC-MS-MS) and reverse phase HPLC (RP-HPLC). For LC-MS-MS analysis, a Waters Acquity I Class – Xevo TQD system using an Acquity BEH C18 column (50x2.1mm, 1.7 µm) at 40°C was used. The mobile phase at a flow of 600 µL/min was composed by two solvents: ammonium acetate 10 mM (Solvent A, spontaneous pH) and acetonitrile (CH_3CN) with 0,1% HCOOH (Solvent B) (UPLC gradient of B= 0% at time 0.98% from minute 2.00 to 2.50 then 0% from minute 2.60) and its flow was 600 µL/min.

Samples were prepared by diluting them 100 times in MeOH and then 100 times in a solution of MeOH containing the internal standards, then 1 µL of each sample was injected in the system.

The RP-HPLC analysis were performed after dilution with the mixture acetonitrile/water 45/55 v/v using a Agilent HPLC system (Agilent 1100 Series) equipped with a C18 column (Kinetex 5u C18, 100 A, Phenomenex, and UV detector at λ = 280 nm. The chromatographic conditions were as follows: solvent A 0.1% Trifluoroacetic Acid (TFA) in water and solvent B 0.1% TFA in acetonitrile; 0–2 min: 0%–20% B, 2–6 min: 20%–45% B, 6–10 min: 45%-75% B, 10–15 min: 75%–0%. Flow rate: 1.0 mL/min at room temperature.

In all cases, R^2 values for both ETH and Booster were higher than 0.99. From the obtained data the DL and EE were calculated as shown in equation 1 and 2, respectively. The DL can be defined as the mass fraction of a NP that is composed of drug, while the EE can be considered as the fraction of drug effectively encapsulated into the NPs compared with the amount that was initially added during the particles preparation [8,9].

(1) $$DL(\%) = \frac{(mg\ of\ encapsulated\ drug^{**})}{(mg\ of\ polymer)} \times 100$$

(2) $$EE(\%) = \frac{(mg\ of\ encapsulated\ drug^{**})}{(mg\ of\ drug\ initially\ added\ to\ the\ formulation)} \times 100$$

[***The amount of encapsulated drug has been calculated with a direct or indirect estimation, depending on the sample fraction considered]*

Bacteria and macrophages

M. tuberculosis H37Rv strain constitutively expressing the green fluorescent protein (H37Rv-GFP) was used as a reporter for the replication assay. Bacteria were cultured at 37 °C for 16 days in complete 7H9 medium containing 0.5 % glycerol (50405, Euromedex), 10% Middelbrook oleic acid-albumin-dextrose-catalase (OADC) (211886, Becton Dickinson), 0.05% Tween 80 (2002A, Euromedex) and 50 µg/mL hygromycin B (10687010, Invitrogen). In the day of the experiment, *M. tuberculosis* were washed with D-PBS Ca- Mg- (14190169, LifeTechnologies) 3 times with centrifugation at 5,000 RPM for 5 minutes and centrifuged at 700 RPM for 2 minutes to remove clumped bacteria. Bacteria were resuspended in RPMI-1640 + glutamax (61870044, LifeTechnologies) containing 10 % heat inactivated fetal bovine serum (FBS) (10270106, Gibco) and titrated by measuring the optical density at 600 nm.

Mouse macrophage RAW 264.7 (ATCC # TIB-71) were maintained at 37°C in RPMI-1640 + glutamax containing 10% FBS and were passed 3 times per week and used before passage number 7. Macrophages were harvested by using Versene (15040033, LifeTechnologies).

Assay plate preparation

ETH, BDM41906 and INH were diluted in DMSO (34943, Sigma-Aldrich) to 10 mg/mL and were dispensed in Echo-qualified 384-well low dead volume source plates (Labcyte). Echo 550 Series Liquid Handler (Labcyte) was used to transfer precise volumes between 5 and 500 nL from the Echo-qualified plate to the 384-well clear-bottom polystyrene assay plates (781091, Greiner Bio-One) by using sound waves. All the conditions were backfilled with DMSO until 500 nL. Prior to addition of cells, 4.5 µL of cell medium were dispensed in the Echo-dispensed wells.

NPs loaded with ETH or with the [ETH:Booster] pair were diluted in water to 0.2 mg/mL of ETH. 2-fold serial dilutions of the mother solution in a final volume of 100 µL were performed in sterile MilliQ water in a 384 deep well “diamond plate” (P-384-120SQ-C-S, Axygen) in order to obtain a dose-response curve. Posteriorly, 5 µL of the NPs were dispensed in the 384-well assay plates.

Extracellular *in vitro* assay

Bacteria were diluted at 2×10^6 bacteria/mL using complete 7H9 medium and 45 µL/well of bacterial suspension were added in 384-well assay plates. After 5 days incubation at 37°C, 5% CO_2, extracellular plates were read using a fluorescence reader (Victor X3, Perkin Elmer) at excitation/emission of 485/535 nm for 0.1 seconds/well with a small emission aperture and CW-lamp energy of 50,000. The read-out, relative fluorescence units (RFU), versus the ETH concentration was then plotted using GraphPad Prism 5.0 software and the concentration required to inhibit 50% of the bacterial replication (IC_{50}) was calculated by nonlinear regression analysis using the equation for a sigmoidal dose-response curve with variable slope.

Intramacrophage *in vitro* assay

For intracellular assay, bacteria were mixed with RAW 264.7 macrophages to prepare a suspension at 5×10^5 cells/mL and 1×10^6 bacteria/mL (multiplicity of infection 2) in RPMI-1640 + glutamax containing 10% FBS. After 2 hours of infection at 37°C with shaking (120 rpm), infected cells were washed with RPMI-1640 + glutamax containing 10% FBS by centrifugation at 1,100 rpm for 5 minutes. The remaining extracellular bacilli from the infected cell suspension were killed by a 1 hour 50 µg/mL amikacin (A2324-5G, Sigma) treatment and then washed twice with RPMI-1640 + glutamax containing 10% FBS. Finally, 45 µL/well of cellular suspension was added in the 384-well assay plate and incubated during 5 days at 37°C, 5% CO_2. Macrophages were then stained with 5 µM Syto 60 (S11342, Molecular probes) dye for 1 hour, followed by plate sealing, imaging acquisition and data analysis.

Image acquisition

Confocal images were recorded on an automated fluorescent ultra-high-throughput microscope Opera (Perkin Elmer). This microscope is based on an inverted microscope architecture that allows imaging of cells cultivated in 96- or 384-well microplates. Images were acquired with a 20X- water immersion objective (NA 0.70). A double laser excitation (488-nm and 640-nm) and dedicated dichroic mirrors were used to record green fluorescence of mycobacteria and red fluorescence of the macrophages on two different cameras, respectively. A series of 6 pictures at the center of each well were taken and each image was then analyzed using Columbus system version 2.5.1 as previously described [10] to extract the bacterial area and the number of cells.

Intramacrophage assay data analysis

The intracellular bacterial area was normalized with the negative control DMSO (0% inhibition) and the positive control INH at a concentration of 1 µg/mL (100 % inhibition) by converting it into a percentage of bacterial replication inhibition (% inhibition). % inhibition was calculated as shown in the equation 3.

$$(3)\ \%\,inhibition = \left(1 - \frac{\text{Test bacterial area} - \text{INH bacterial area}}{\text{DMSO bacterial area} - \text{INH bacterial area}}\right) \times 100$$

This formula was used to normalize the “test bacterial area” from different experiments, in order to decrease potential bias in the results due to differences in the % of infection and bacterial growth between experiments. For each compound, a plot of % inhibition versus the ETH concentration was determined with GraphPad Prism 5.0 software and the IC_{50} was calculated in the same way as in the extracellular assay.

In vivo **efficacy**

6-week old Balb/C female mice were purchased from Janvier (Le Genest-Saint-Isle, France) and were maintained in the animal house facility of the Pasteur Institute of Lille, France (Agreement B59-350009). The project received ethical approval by French Committee on Animal Experimentation and all experiments were performed in accordance with relevant guidelines and regulations.

Lung histology

Infected or uninfected 8-week-old mice were divided in groups and endotracheally administered with water (vehicle) or pCD NPs (Fig 5~~a~~, Fig.6).
At the determined end-point mice were euthanized, lungs were harvested and soaked in 4% formaldehyde (10% formalin solution, neutral buffered, HT501128, Sigma-Aldrich) for 24 hours, before being embedded in paraffin. Tissues were sliced with microtome and 5 µm sections were stained with Hematoxylin-Eosin (H-E) for light microscopy examination for anatomopathology.

Flow cytometry

Harvested lungs were cut into small pieces and incubated for 1 hour at 37°C with a mix of DNAse I (100 µg/ml, , Sigma-Aldrich) and collagenase (1.6 mg/ml, Roche) 400 U/ml. Lung cells were washed and filtered before being incubated with saturating doses of purified 2.4G2 (anti-mouse Fc receptor, ATCC) in 200 µl PBS 0.2 % BSA 0.02 % NaN3 (FACS buffer) for 20 minutes at 4°C to prevent antibody binding on the Fc receptor. Various fluorescent mAb combinations in FACS buffer were used to stain 3-5x106 cells. Acquisitions were done on FACScantoII cytofluorometer (Becton Dickinson) with the following mAbs from BD Biosciences: Fluorescein (FITC)-coupled HL3 (anti-CD11c), FITC-coupled 145-2C11 (anti-CD3), APC-coupled RB6-8C5 (anti-GR1), phycoérythrine (PE)-coupled RM4-5 (anti-

CD4), PE-coupled E50-2440 (anti-SIGLEC-F), APC-coupled BM8 (anti-F4/80). APC-eF780-coupled M1/70 (antiCD11b) was purchased from eBiosciences and fixable viability dye eFluor 506 (eBiosciences) was used to gate viable cells.

Efficacy studies

8-week-old mice (4 mice per group) were inoculated with *M. tuberculosis* H37Rv via the intranasal route to 10^6 bacteria in the lungs at day 0. On day 7, 9 and 11 after infection, NPs or INH were administered to mice using a Microsprayer® (MicroSprayer® Aerosolizer – Model IA-1C-M and FMJ-250 High Pressure Syringe, Penn Century Inc., Wyndmoor, PA). For the 1-week treatment and the 2-week treatment, administrations were on day 7, 9, 11 and on day 7, 9, 11, 14, 16, 18 respectively. Body weight were recorded after each treatment (Fig. 5b).. To assess the reproducibility of NPs administration through the MicroSprayer®, the delivered doses of NP suspensions were collected in glass vials after each spray and were accurately weighed. Then the amount of the delivered drug was quantified by HPLC as already described.

The protocol to administer the NPs in mice was adapted from a previously reported one [11]. Briefly, mice were placed in isoflurane chamber (Aerrane®, Baxter SAS, France). Then, one mouse was placed on the back on a platform (Mouse Intubation Platform – Model MIP, Penn Century Inc., Wyndmoor, PA) with isoflurane mask and hanging on its teeth. The tongue was pulled out by a tweezer and a laryngoscope (Small Animal Laryngoscope for mouse – Model LS-2-M, Penn Century Inc., Wyndmoor, PA) was used to see the trachea and 50µL of suspensions were delivered inside the lung allowing to aerosolize 50 µL suspensions inside the lungs.

At day 14 or 21, mice were euthanized and lungs were homogenized with MM300 bead beater (Retsch) and eight ten-fold serial dilutions were plated onto 7H11 agar plates supplemented with 10% OADC. CFUs were determined after a three-week growth. Represented data are mean values ± standard deviation of one representative experiment from three independent experiments. Results for each independent experiment were summarized in **Supplementary Table S2**. Statistics were performed using Student's t-test and one-way ANOVA analysis. Same p-values for in vivo experiments were obtained with the two tests. *: $p<0.1$, **: $p<0.01$,***: $p<0.001$.

Supplementary Video S1 – NTA video of a NP suspension obtained by nanoemulsion. NTA allows tracking each NP individually to determine its mean hydrodynamic diameter and the concentration of the sample. Indeed, the Nanosight instrument is able to track the Brownian motion of NPs, in order to determine its diffusion coefficient (D_t) and consequently calculate, using Stokes-Einstein equation the sphere-equivalent hydrodynamic diameter. The NP suspensions were diluted by a factor of 10^4 to fit the instrument requirements and to allow the particle to move freely according to their Brownian motion. More than 2000 NPs are followed in each experiment.

References

1 Daoud-Mahammed, S. *et al.* Cyclodextrin and polysaccharide-based nanogels: entrapment of two hydrophobic molecules, benzophenone and tamoxifen. *Biomacromolecules* **10**, 547-554, doi:10.1021/bm801206f (2009).

2 Bouchemal, K., Couvreur, P., Daoud-Mahammed, S., Poupaert, J. & Gref, R. A comprehensive study on the inclusion mechanism of benzophenone into supramolecular nanoassemblies prepared using two water-soluble associative polymers. *Journal of Thermal Analysis and Calorimetry* **98**, 57, doi:10.1007/s10973-009-0452-2 (2009).

3 Daoud-Mahammed, S. *et al.* Spontaneous association of hydrophobized dextran and poly-beta-cyclodextrin into nanoassemblies. Formation and interaction with a hydrophobic drug. *Journal of colloid and interface science* **307**, 83-93, doi:10.1016/j.jcis.2006.10.072 (2007).

4 Flipo, M. *et al.* Ethionamide boosters. 2. Combining bioisosteric replacement and structure-based drug design to solve pharmacokinetic issues in a series of potent 1, 2, 4-oxadiazole EthR inhibitors. *Journal of medicinal chemistry* **55**, 68-83 (2011).

5 Gref, R. *et al.* New self-assembled nanogels based on host-guest interactions: characterization and drug loading. *J Control Release* **111**, 316-324, doi:10.1016/j.jconrel.2005.12.025 (2006).

6 Othman, M. *et al.* A comprehensive study of the spontaneous formation of nanoassemblies in water by a "lock-and-key" interaction between two associative polymers. *J Colloid Interface Sci* **354**, 517-527 (2011).

7 Bilati, U., Allémann, E. & Doelker, E. Nanoprecipitation versus emulsion-based techniques for the encapsulation of proteins into biodegradable nanoparticles and process-related stability issues. *Aaps Pharmscitech* **6**, E594-E604 (2005).

8 Kumar, G. *et al.* In vitro physicochemical characterization and short term in vivo tolerability study of ethionamide loaded PLGA nanoparticles: potentially effective agent for multidrug resistant tuberculosis. *J Microencapsul* **28**, 717-728, doi:10.3109/02652048.2011.615948 (2011).

9 Ankrum, J. A. *et al.* Engineering cells with intracellular agent–loaded microparticles to control cell phenotype. *Nat. Protocols* **9**, 233-245, doi:10.1038/nprot.2014.002 (2014).

10 Queval, C. J. *et al.* A microscopic phenotypic assay for the quantification of intracellular mycobacteria adapted for high-throughput/high-content screening. *J Vis Exp*, e51114, doi:10.3791/51114 (2014).

11 Bivas-Benita, M., Zwier, R., Junginger, H. E. & Borchard, G. Non-invasive pulmonary aerosol delivery in mice by the endotracheal route. *Eur J Pharm Biopharm* **61**, 214-218 (2005).
